# Within study comparisons and risk of bias in international development: Systematic review and critical appraisal

**DOI:** 10.1002/cl2.1027

**Published:** 2019-07-26

**Authors:** Paul Fenton Villar, Hugh Waddington

**Affiliations:** ^1^ School of International Development University of East Anglia Norwich UK; ^2^ International Initiative for Impact Evaluation (3ie) and London School of Hygiene and Tropical Medicine London UK

## Abstract

**Background:**

Many systematic reviews incorporate nonrandomised studies of effects, sometimes called quasi‐experiments or natural experiments. However, the extent to which nonrandomised studies produce unbiased effect estimates is unclear in expectation or in practice. The usual way that systematic reviews quantify bias is through “risk of bias assessment” and indirect comparison of findings across studies using meta‐analysis. A more direct, practical way to quantify the bias in nonrandomised studies is through “internal replication research”, which compares the findings from nonrandomised studies with estimates from a benchmark randomised controlled trial conducted in the same population. Despite the existence of many risks of bias tools, none are conceptualised to assess comprehensively nonrandomised approaches with selection on unobservables, such as regression discontinuity designs (RDDs). The few that are conceptualised with these studies in mind do not draw on the extensive literature on internal replications (within‐study comparisons) of randomised trials.

**Objectives:**

Our research objectives were as follows:

Objective 1: to undertake a systematic review of nonrandomised internal study replications of international development interventions.

Objective 2: to develop a risk of bias tool for RDDs, an increasingly common method used in social and economic programme evaluation.

**Methods:**

We used the following methods to achieve our objectives.

Objective 1: we searched systematically for nonrandomised internal study replications of benchmark randomised experiments of social and economic interventions in low‐ and middle‐income countries (L&MICs). We assessed the risk of bias in benchmark randomised experiments and synthesised evidence on the relative bias effect sizes produced by benchmark and nonrandomised comparison arms.

Objective 2: We used document review and expert consultation to develop further a risk of bias tool for quasi‐experimental studies of interventions (ROBINS‐I) for RDDs.

**Results:**

Objective 1: we located 10 nonrandomised internal study replications of randomised trials in L&MICs, six of which are of RDDs and the remaining use a combination of statistical matching and regression techniques. We found that benchmark experiments used in internal replications in international development are in the main well‐conducted but have “some concerns” about threats to validity, usually arising due to the methods of outcomes data collection. Most internal replication studies report on a range of different specifications for both the benchmark estimate and the nonrandomised replication estimate. We extracted and standardised 604 bias coefficient effect sizes from these studies, and present average results narratively.

Objective 2: RDDs are characterised by prospective assignment of participants based on a threshold variable. Our review of the literature indicated there are two main types of RDD. The most common type of RDD is designed retrospectively in which the researcher identifies post‐hoc the relationship between outcomes and a threshold variable which determines assignment to intervention at pretest. These designs usually draw on routine data collection such as administrative records or household surveys. The other, less common, type is a prospective design where the researcher is also involved in allocating participants to treatment groups from the outset. We developed a risk of bias tool for RDDs.

**Conclusions:**

Internal study replications provide the grounds on which bias assessment tools can be evidenced. We conclude that existing risk of bias tools needs to be further developed for use by Campbell collaboration authors, and there is a wide range of risk of bias tools and internal study replications to draw on in better designing these tools. We have suggested the development of a promising approach for RDD. Further work is needed on common methodologies in programme evaluation, for example on statistical matching approaches. We also highlight that broader efforts to identify all existing internal replication studies should consider more specialised systematic search strategies within particular literatures; so as to overcome a lack of systematic indexing of this evidence.

## 1 INTRODUCTION

Many systematic reviews include studies that use nonrandomised causal inference, hereafter called nonrandomised studies, and sometimes called quasi‐experiments (QEs; e.g., Bärnighausen, Røttingen, Rockers, Shemilt, & Tugwell, 2017; Shadish, Cook, & Campbell, 2002) or natural experiments (Dunning, 2012).^1^ For example, Konnerup and Kongsted (2012) found that half of the systematic reviews published in the Campbell Library up to 2012 included nonrandomised studies. The inclusion of nonrandomised studies in Campbell reviews is increasing: 81% of reviews published between 2012 and 2018 included such studies. The inclusion of nonrandomised studies in reviews is justified by the lack of randomised study evidence for specific interventions, for example where randomisation is not considered feasible (Wilson, Gill, Olaghere, & McClure, 2016), or ethical (e.g., mortality outcomes), or to improve external validity such as in measuring long‐term effects (Welch et al., 2016).^2^ Occasionally it is stated that these studies might produce unbiased estimates (e.g., De La Rue, Polanin, Espelage, & Piggot, 2014).^3^ However, it is not clear whether nonrandomised studies typically produce comparable treatment effect estimates to unbiased estimates produced by well‐conducted randomised controlled trials (RCTs), either in expectation or in practice.

There are two main types of study for quantitative causal inference (Imbens & Wooldridge, 2009):

(a) Those which account for unobservable confounding by design, either through knowledge about the method of allocation or in the methods of analysis used, referred to as “selection on unobservables”. These include RCTs and nonrandomised approaches such as difference studies (e.g., the difference in differences and fixed effects analysis), instrumental variables (IVs) estimation, interrupted time series (ITS) and regression discontinuity designs (RDDs).
(b) Those with selection on observables only, including nonrandomised studies that control directly for confounding in adjusted analysis (e.g., statistical matching, analysis of covariance (ANCOVA), multivariate regression).

Nonrandomised studies modelling selection on unobservables are considered more credible in theory (Dunning, 2012; Imbens & Wooldridge, 2009; Shadish et al., 2002). But many design and analysis factors determine the extent to which nonrandomised studies (with selection on unobservables or observables) are biased in practice, and by how much.

There are two main ways to empirically measure the magnitude of bias in nonrandomised studies (Bloom et al., 2002). One is in “cross‐study comparison” of groups of randomised and nonrandomised studies, usually done in systematic review and meta‐analysis. For example, evidence from meta‐analyses of programmes in low‐ and middle‐income countries (L&MICs) suggests nonrandomised studies with credible means of control for confounding (including difference in differences, IVs and statistical matching) can produce the same pooled effects as RCTs, although potentially with less precision (Waddington et al., 2017). Lipsey and Wilson (1993), in a meta‐analysis of meta‐analyses containing a very broad range of study designs, found the point estimates calculated from meta‐analyses of nonrandomised trials were on average virtually identical to those from RCTs. However, there are doubts about the validity of cross‐study comparisons in quantifying bias, even when these studies find zero differences in treatment effects across randomised and nonrandomised studies on average. They are usually based on indirect comparisons from different underlying populations, and it is argued that there is no theoretical reason why one should expect any differences to cancel out on average (Cook, Shadish, & Wong, 2008).^4^

The second, and conceptually preferred, approach is the “internal replication study”, which assesses the validity of nonrandomised comparison group designs, with reference to a “benchmark” study that is thought to be unbiased. The most rigorous designs use data from the same underlying treatment population, hence they are also referred to as “within‐study comparisons” (Bloom et al., 2002; Glazerman, Levy, & Myers, 2003). These studies benchmark the effect sizes obtained using nonrandomised comparison group designs, to estimates from designs that are in expectation unbiased, usually RCTs. It is important that the treatment sample used in the benchmark and replication studies overlap, because of potential differences in treatment effect parameter—for example, the average treatment effect (ATE) causal estimand from an RCT versus the local average treatment effect (LATE) estimand from a RDD—over and above errors to due sampling or bias (Duvendack et al., 2012).

Evidence from internal replication studies suggests that nonrandomised studies in which the method of treatment assignment is known or credibly modelled at baseline, can produce very similar findings in direct comparisons with RCTs (Cook et al., 2008; Hansen, Klejnstrup, & Andersen, 2013). However, when inappropriately designed or executed, they are likely to yield biased effect size estimates (Cook et al., 2008; Glazerman et al., 2003; Pirog, Buffardi, Chrisinger, Singh, & Briney, 2009). The extent of bias is likely to depend on the design of the evaluation, how the evaluation design is implemented and the quality of analysis and reporting.

Work is, therefore, needed to quantify the biases arising in different nonrandomised studies and assess the extent that these relate to estimates of bias produced in critical appraisal. This includes validating risk of bias tools for studies included in systematic reviews.

We have attempted to address this research gap by systematically reviewing internal replication studies of benchmark randomised experiments in international development, and extending a risk of bias tool for regression discontinuity (RD), a popular nonrandomised study design used in international development programme evaluation and increasingly incorporated in systematic reviews of that evidence. The remainder of the document is structured as follows. Section 2 presents the study objectives. Section 3 presents the results of the systematic review. Section 4 presents proposed approach to assessing risk of bias for RDDs. The final section presents implications for systematic review practice and research.

## 2 RESEARCH OBJECTIVES AND APPROACH

Our objectives were to conduct a systematic review of internal replication studies in international development, and further develop and pilot a tool to assess risk of bias for RDDs, an increasingly popular method of causal inference in international development research.

- Research objective 1: systematic review of internal replication studies in international development. This included:
  a.  Review of existing narrative reviews of internal replication studies (e.g., Cook et al., 2008; Hansen et al., 2013; Wong, Valentine, & Miller‐Bains, 2017) and meta‐analyses of these studies (e.g., Chaplin et al., 2018; Glazerman et al., 2003).
  b.  Systematic electronic and hand‐searches for internal replication studies in international development.
  c.  Critical appraisal (risk of bias assessment) in benchmark trials.
  d. Calculation of standardised bias estimates and narrative analysis of differences in effect sizes between the benchmark and nonrandomised QE study arms.
- Research objective 2: development of a risk of bias tool in nonrandomised studies of interventions (ROBINS‐I) for assessing risk of bias in RDDs. This included:
  a.  Review of methods used to assess bias in nonrandomised studies in Campbell systematic reviews.
  b.  Reviewing literature on RDD and developing the tool.

## 3 SYSTEMATIC REVIEW OF INTERNAL REPLICATION STUDIES IN INTERNATIONAL DEVELOPMENT

Internal replication studies, also called “within study comparisons”, are studies which compare a nonrandomised comparison group with an unbiased “causal benchmark” study. They have been conducted in the social sciences since the 1980s, following an internal replication of the randomised evaluation of the National Supported Work Demonstration programme in the United States (Lalonde, 1986). We aimed to identify the universe of internal replication within‐study comparisons of social and economic programmes in the social sciences in L&MICs. In this section, we present a review of existing literature reviews, including categories of, and sources of bias in, internal replication studies, and results of systematic searches and data collection from internal replication studies in L&MICs.

### 3.1 Literature review

Table 1 presents a list of known existing reviews of internal replication studies. Some are of particular literatures, for example, studies of labour market programmes (Glazerman et al., 2003) and education (Wong et al., 2017). Others cover particular methodological designs, such as RDD (Chaplin et al., 2018; Cook & Wong, 2008) and propensity score matching (PSM; Shadish, 2013), or map internal replication designs (Wong and Steiner, 2016). Only one known review is dedicated to evidence from social and economic development programmes in L&MICs (Hansen et al., 2013).

**Table 1:** Existing reviews of within‐study comparisons by publication date

Authors	Title	Publisher
Bloom, Michalopoulos, Hill and Lei (2002)	Can nonexperimental comparison group methods match the findings from a random assignment evaluation of mandatory welfare‐to‐work programs?	MDRC
Glazerman, Levy and Myers (2002)	Nonexperimental replications of social experiments: A systematic review	Mathematica Policy Research, Inc.
Glazerman et al. (2003)	Nonexperimental versus experimental estimates of earnings impacts	The Annals of the American Academy
Cook and Wong (2008)	Empirical tests of the validity of the regression discontinuity design	Institute for Policy Research Northwestern University Working Paper Series
Cook et al. (2008)	Three conditions under which experiments and observational studies produce comparable causal estimates: New findings from within‐study comparison	Journal of Policy Analysis and Management
Pirog et al. (2009)	Are the alternatives to randomized assignment nearly as good? Statistical corrections to nonrandomized evaluations	Journal of Policy Analysis and Management
Shadish and Cook (2009)	The renaissance of field experimentation in evaluating interventions	Annual Review of Psychology
Shadish et al. (2012)	A case study about why it can be difficult to test whether propensity score analysis works in field experiments	Journal of Methods and Measurement in the Social Sciences
Hansen et al. (2013)	A comparison of model‐based and design‐based impact evaluations of interventions in developing countries	American Journal of Evaluation
Shadish (2013)	Propensity score analysis: Promise, reality and irrational exuberance	Journal of Experimental Criminology
Cook (2014)	Testing causal hypotheses using longitudinal survey data: A modest proposal for modest improvement	Mathematica Policy Research, Inc.
Steiner and Wong (2016)	Assessing correspondence between experimental and nonexperimental results in within‐study‐comparisons	EdPolicyWorks Working Paper
Wong and Steiner (2016)	Designs of empirical evaluations of nonexperimental methods in field settings	EdPolicyWorks Working Paper
Jaciw (2016)	Assessing the accuracy of generalized inferences from comparison group studies using a within‐study comparison approach: The methodology	Evaluation Review
Wong et al. (2017)	Empirical performance of covariates in education observational studies	Methodological Studies
Chaplin et al. (2018)	The internal and external validity of the regression discontinuity design: A meta‐analysis of 15 within‐study‐comparisons	Policy Analysis and Management

Hansen et al. (2013) surveyed four studies, involving two cluster‐randomised conditional cash transfer programmes in Mexico and Nicaragua^5^ and an individually randomised lottery balloting permanent migration visas in Tonga.^6^ One study in Mexico examined the correspondence of estimates from an RDD analysis with estimates from a cluster‐randomised controlled trial (Buddelmeyer & Skoufias, 2004). The remaining studies examined the correspondence of difference‐in‐difference (DID), matching and IV techniques (Diaz & Handa, 2006; Handa & Maluccio, 2010; McKenzie, Stillman, & Gibson, 2010). Findings from this review highlighted that across the four studies the nonrandomised estimators did offer instances where correspondence with randomised estimates was high (suggesting nonrandomised estimators can provide unbiased estimates), but that this was not always the case. In particular, in the context of the evaluation of development interventions, nonrandomised studies were more relevant in contexts where self‐selection is negligible and the selection process is simple or well understood.

However, Hansen et al. (2013) did not use systematic approaches for study identification or formal critical appraisal of studies. In fact, few of the reviews in this body of literature appear to have been conducted systematically (White and Waddington 2012; Waddington et al 2012). Exceptions include a review by Wong et al. (2017), who report a systematic search strategy, and meta‐analyses by Glazerman et al. (2003) and Chaplin et al. (2018), although concerns regarding the completeness of their search strategies are noted by the authors themselves. Glazerman et al. (2003) indicate that electronic searches failed to comprehensively identify many known studies. This was due to the lack of a common language to define an internal replication study. Furthermore, it is not uncommon that such studies feature as undefined empirical demonstrations in new methods papers or as a secondary piece of analysis in a broader study. Similar problems were also noted by Chaplin et al. (2018), who state that despite having searched broadly “we cannot even be sure of having found all past relevant studies” (p. 424).

Nevertheless, as the first meta‐analysis synthesising this body of evidence, Glazerman et al. (2003) identified 12 studies on job training and employment services^7^ where the dependent variable was earnings. All studies originated in high‐income contexts, based on data collected on interventions in the United States and one in Norway; three‐quarters of the interventions and data collection were concluded in the 1970s and 1980s. The analysis examined study findings from a range of different methodological approaches (including cross‐section, panel and DIDs regression, statistical matching and selection models). It concluded that nonrandomised methods rarely replicated experimental estimates and the absolute magnitude of the differences was often quite large.^8^

Non‐systematic qualitative updates of this review by Cook et al. (2008) and Pirog et al. (2009) later highlighted that with “careful execution” nonrandomised estimators can recreate randomised estimates, and nonrandomised estimates based on inappropriately designed estimation procedures were associated with larger bias coefficients (Cook et al., 2008).

Reviews by Cook and Wong (2008), Cook et al. (2008) and Pirog et al. (2009) later expanded the scope of nonrandomised estimators examined, including ITS and RDD. Drawing from a limited base of evidence, they suggested that an ITS study could also create similar results to an RCT. Similarly, they concluded that studies using RDD provide estimates that are comparable to an RCT estimate when it is made for observations close to the discontinuity (or “cut‐off”) in the assignment (or “forcing”) variable.

Building on these findings, Chaplin et al. (2018) further assessed the statistical correspondence of 15 internal replication studies with an RDD approach (including two studies based on data collected on programmes in L&MICs) using meta‐analysis. They reported that the average of the difference between RCT and RDD estimates around the discontinuity is close to zero (approximately 0.1 standard deviations) and that the variability of results was also generally quite low. However, they warned that researchers should not assume based on these findings that individual RDD estimates will necessarily be near zero. They suggested factors such as larger samples, using nonparametric tests and the choice of bandwidths may prove important in determining the degree of bias in an individual RDD estimate.

Further reviews have included mapping internal replication designs and describing different measures of bias (or correspondence; e.g., see Jaciw, 2016; Wong & Steiner, 2016). Wong and Steiner (2016) describe broad categories of internal replication studies, including independent, synthetic, simultaneous and multisite simultaneous designs (Table 2).

**Table 2:** Definitions of within‐study comparison designs

Within‐study comparison type	Description
1. Independent design	Also known as the “four‐arm” design, participants are randomly assigned into benchmark and nonrandomised arms. Participants in the benchmark arm are randomly assigned again into treatment/control conditions. Participants in the nonrandomised arm self‐select or are selected by a third party into a preferred treatment option.
2. Synthetic design	The researcher begins with data from an RCT and then constructs a nonrandomised study by simulating a selection process and removing information from the RCT treatment and/or control group to create nonequivalent groups.
3. Simultaneous design	Observations from an overall population select (or are selected) to participate in the benchmark study. In the benchmark study, participating observations are randomly assigned into treatment conditions, and the estimated treatment effect serves as the causal benchmark result for evaluating the nonrandomised approach. For the nonrandomised arm of the comparison, the researcher compares the RCT treatment units with comparisons from a sample of the population that did not participate in the RCT. Here we note a special type of this design is also referred to as a ‘tie‐breaker’ design. Further described by Chaplin et al. (2018), the initial selection into the benchmark study is determined by an eligibility criteria. This then commonly enables researchers to form a comparison between the RCT regression discontinuity design estimates.
4. Multi‐site‐simultaneous design	Beginning with a multi‐site RCT in which randomisation occurs within sites (sites are purposefully selected but randomisation to a treatment condition occurs within each site), the nonrandomised arm is then constructed by comparing average outcomes from an RCT treatment group in one site to an RCT control case from another site. Here, the composition of units will differ from site to site and not be random due to the nonrandom selection of sites.

Abbreviation: RCT, randomised controlled trial.

*Source*: Definitions adapted from Wong and Steiner (2016).

However, authors such as Smith and Todd (2005, p. 306) warn against “searching for ‘the’ nonexperimental estimator that will always solve the selection bias problem inherent in nonexperimental evaluations”. Instead they argue research should seek to map and understand the contexts that may influence studies’ degrees of bias. For instance, Chaplin et al. (2018) consider that their review says little about instances when an experiment is logistically very difficult to implement, or noncompliance is likely to be large. Hansen et al. (2013) note the potential importance of the type of dependent variable examined in studies, suggesting simple variables (such as binary indicators of school attendance) may be easier to model relative to more complex outcome variables (such as consumption expenditure or earnings). Meanwhile, Glazerman et al. (2003) find factors such as the source of data, the quality of control variables and evidence of statistical robustness tests are related to the magnitude of estimator bias.

Jaciw (2016) provides a broader review of characteristics associated with bias in internal replication studies. The author notes that studies have found the comparison groups’ geographic proximity, the richness of background controls, use of baseline outcomes as control variables and the complexity of outcome variables to be related with the degree of bias among nonrandomised estimators. Meanwhile, investigating best practices for selecting covariates in education research, Wong et al. (2017) synthesise results from 12 internal replication studies (all from high‐income countries) where the dependent variable included a standardised reading or math test score. Similarly, they describe baseline outcomes, geographic proximity and the richness of control variables are important factors that may determine the magnitude of bias among nonrandomised estimators. They also note where nonrandomised studies simply rely on a set of demographic variables, or those that prioritise local matching when local comparisons are not comparable to treated cases, they will rarely replicate similar estimates to RCTs. In Table 3 we summarise the list of factors which internal replication studies hypothesise may be associated with bias in nonrandomised studies.

**Table 3:** Factors associated with bias in internal replication studies

Study Characteristic	Description
Methods and procedures	Most common is the use of internal replication studies to examine whether a method or a new technique can reduce the bias of nonrandomised studies (e.g., Hainmueller, 2012; Diamond and Sekhon, 2012). This also includes more nuanced assessments examining the effects of specific procedures. For example, examining bandwidth size or use of calipers in RDD (e.g., Green et al., 2009; Gleason et al., 2012; Wing and Cook, 2013; Moss et al., 2014)
Preintervention outcome data	Reasons for assuming the inclusion of preintervention outcomes in nonrandomised analysis can reduce bias in nonrandomised studies include that preintervention outcomes will likely be highly correlated with unobserved determinants of the outcome variable. Internal study replications have examined whether including preintervention outcome data in model specifications may help to reduce and minimise the confounding effects of at least some of the unobserved factors determining observations outcomes post‐intervention (e.g., Lalonde, 1986; Smith and Todd 2005; Zhao, 2006; Steiner et al. 2010; Bifulco, 2012; Wong et al., 2017; Fortson et al., 2014; Calónico and Smith, 2017)
Length of preintervention history	In an extension to studies’ attempts to understand the extent that the inclusion of preintervention outcome data may reduce bias (see above), some research explores the effects of having preintervention data from numerous data points (e.g., Michalopoulos et al., 2004; Hallberg et al., 2016)
Types of outcomes	Some research now exists comparing the efficacy of quasi‐experimental methods to replicate RCT estimates when using different types of outcome variables. For example, Diaz and Handa (2006) and Handa and Maluccio (2010) contrast levels of bias following applications of these methods to a broad range of common development outcomes; including student enrolment, child labour, and food expenditure. Liebman et al. (2004) also compare findings using education, behavioural, and physical health outcomes
Richness and types of variable data	A number of internal study replications distinguish the effects that data availability may have on bias in nonrandomised studies. This includes comparing bias in parsimonious specifications of models (e.g., Lalonde, 1986; Smith and Todd, 2005; Gordon et al., 2016), as well as examining constructs of data types. For example, Cook et al. (2009), Cook and Steiner (2010) and Steiner et al. (2010) examine the effects of including or excluding variables related to areas such as psychological predisposition, demographics, topic preferences and so forth, and Hallberg et al. (2013) examined applying student and school level characteristics to their specifications. Calónico and Smith (2017) and Anderson and Wolf (2017) also examine whether the inclusion of common demographic data in model specifications is important in reducing bias
Geographic markets and aggregate conditions	Characteristics of local environments, such as economies and labor markets, may be pivotal in explaining the differences in the development of particular areas and their constituents. Here studies have examined the effects of adjusting model specification for aggregate conditions (such as the local areas unemployment rate—see Hill et al., 2004). Alternatively, others have considered limiting the sample that the comparison group is drawn from to the local location (such as neighbouring counties—see Lee, 2006)
Higher order terms and interacted variables	Increasingly internal replication studies have begun to experiment using higher order terms (such as cubed or squared variables) and interaction terms in model specifications. This reflects that some bias may be derived from the model's mis‐specification of the functional form (e.g., Dehejia and Wahba, 1999; Green et al., 2009; Fortson et al., 2014). Extending on this point, other research has sought to establish and test a means for handling specification uncertainty (e.g., Kitagawa and Muris, 2016)
Use of no‐shows as a comparison	No‐shows are individuals who enrol for a programme, are accepted onto the programme, but for some reason do not participate in it. Debate exists whether this group hosts attractive qualities useful for identifying valid comparisons. For example, they derive from the same geographic locations as programme participants and have undergone the same questioning process during data collection. However, their no‐show status reflects something inherent that distinguishes the individual that may bias an estimator (e.g., Heckman et al., 1997)
Origin of survey data	Another factor that may influence bias includes the origin of the Data set from where the nonrandomised comparison group is derived (Glazerman et al., 2003). This could lead from differences in the way that a survey is administered, the quality of data collection, and the consistency of the measurement of variables (see Heckman et al., 1998, 1997; Agodini and Dynarski, 2004)
Differences in measurement	One of the possible reasons why using survey data from different origins could cause bias in the estimates to increase is that the measurement of variables could be inconsistent across surveys. Handa and Maluccio (2010) provide an example, using adjusted measures of total expenditure as an outcome variable, to show that reported level of bias may be sensitive even to subtle differences in measurement. Fortson et al. (2014) further investigate the magnitude of bias reduction that could be achieved from using errors‐in‐variables models to correct for measurement errors
Management of missing data	A common empirical issue in the social sciences concerns the problem of missing data. For example, this could be where answers or responses to survey questions have not been recorded, provided by the participant, or could not be obtained for a given time period. The way that researchers manage this problem may be another factor that effects bias (Shadish et al., 2008)
Grouped versus individual data	Authors, such as Fraker and Maynard (1987), have compared whether compiling nonrandomised estimates on grouped data (e.g., social security cells), as opposed to individual‐level data, results in greater levels of bias
Target populations	A number of authors have investigated whether quasi‐experimental estimates degree of bias varies across different target populations or subgroups; for example, youth vs. adult and male vs. female (see Heckman et al., 1997; Gritz and Johnson, 2001; Liebman et al. 2004).
Time	Another factor that may affect bias is the length of time after the start of the intervention that the dependent variable is being modelled (see Michalopoulos et al., 2004; Hämäläinen et al., 2008)
Matching Sample Size	Some analysis also considers the size of the nonrandomised sample required to reproduce RCT results. Lee (2006) tests the sensitivity of estimates of bias to the number of observations available for matching in the comparison group (or the ratio of treatment group observations to comparison group observations). Deke and Dragoset (2012) investigate the sample size required for a RDD to replicate the statistical precision of an RCT
Types of discontinuities	Specific to RDD studies, some evidence exists distinguishing bias observed from nonrandomised estimates following the application of different types of discontinuities. For example, Black et al. (2007) consider time, geographic and marginality scoring based discontinuities

Abbreviations: RCT, randomised controlled trial; RDD, regression discontinuity design.

Beyond bias being determined by a particular method, or magnified by a characteristic of a study, another potential source of discrepancy between randomised and non‐randomised designs concerns whether they provide the same causal quantity. In other words, a factor explaining differences in findings across randomised and nonrandomised designs, over and above bias and sampling error, is that they may provide different causal estimands for different treatment populations. For example, Cook et al. (2008) articulate that confounding may occur when comparing an experimental intent‐to‐treat (ITT) estimate with a nonrandomised estimate which computes the average effect on those that receive the treatment of interest (i.e., the ATE on the treated, TOT). Here one issue that arises follows that the average effect reported by the ITT estimator becomes increasingly more conservative as noncompliance rises, making the two estimators not directly comparable.

In another example, Cook et al. (2008) highlight issues arising from estimators derived from LATEs, which are most commonly estimated during the conduct of RDD, to the ATE estimated from an RCT. In this instance, if we are to relax the assumption that the effects of an intervention are homogenous across a population, the size of the LATE effect would be conditional on the point in the population's distribution being assessed (e.g., the point in the distribution that a discontinuity occurs). Again, here the LATE estimate may be an unbiased estimate of the average effect of an intervention amongst the population in immediate proximity to the discontinuity. However, it may also be a very different magnitude to the average effect observed across the entire population that receive the treatment.

### 3.2 Systematic review approach

We used the following approaches for study inclusion criteria, searching and data collection.

*Study design*: Glazerman et al. (2003, p. 65) define a replication study as follows: “researchers estimate a program's impact by using a randomised control group and then re‐estimate the impact by using one or more nonrandomised comparison groups”. We included studies that report treatment effects for a benchmark randomised causal study, alongside treatment effects for a nonrandomly assigned comparison replication. The replicated comparisons could be constructed using any quasi‐experimental method (e.g., DIDs, IVs, statistical matching, RDD).

*Population*: populations in L&MICs were eligible; among general programme participants or lab studies conducted among students. The causal benchmark and comparison study needed to derive from the same study population so as to minimise the possibility of a factor other than study design confounding estimates of bias.

*Intervention and comparator*: studies could be of any social or economic intervention and of any comparison condition (e.g., no intervention, wait‐list, alternate intervention). Where relevant, the causal benchmark and comparison study also needed to derive from the same intervention or comparison condition, and time period.

*Outcome*: studies could be of any outcome variable, provided the outcome was the same for the benchmark control and replication comparison groups.

Studies or study arms were excluded that:

- made between‐study comparisons (with no overlap in treatment group samples for causal benchmark and comparison);
- were based on clinical, biomedical or health care interventions or of populations in high‐income country contexts (e.g., Fretheim et al., 2015; Fretheim, Soumerai, Zhang, Oxman, & Ross‐Degnan, 2013);
- did not use as a causal benchmark a study with randomised or quasi‐randomised allocation, or did not use “real world” data collected from participants. Studies conducting analysis of an artificial or synthetic population (such as Schafer & Kang, 2008), were therefore excluded;
- did not construct the nonrandomised comparison group using quasi‐experimental methods or a natural experiment (such as a retrospective RDD) to account for confounding; a typical example of an excluded comparison would be the use of adjusted regression (ordinary least squares [OLS] or limited dependent variable analysis) applied to observational cross‐section data^9^;
- used a nonrandomised technique to adjust for circumstances where the randomisation process was compromised (e.g., Borland, Tseng, & Wilkins, 2013) or for study attrition (e.g., Grasdal, 2001); and
- compared the predicted estimates of ex‐ante models or general equilibrium models to estimates from ex‐post RCTs (e.g., Leite, Narayan, & Skoufias, 2015; Todd & Wolpin, 2006; Lise & Seitz, 2005).

#### 3.2.1 Search methods

Existing reviews of internal replication studies, such as Glazerman et al. (2003) and Chaplin et al. (2018), note a number of issues related to study identification. In particular, these issues highlight a lack of common language used to systematically index evidence. In order to identify internal replication studies, in this review, we first use a combination of conventional methods; searching electronic academic databases and the bibliographies of identified studies and literature reviews. However, to further identify studies that may not be well indexed in this literature, we supplement this search process using modern citation tracking software to identify studies citing well known reviews of internal replication studies. We also search the repositories of known institutional providers of internal replication studies and 3ie's impact evaluation repository (a specialised database of impact evaluations in international development).

Electronic searches: with the assistance of an information specialist, we searched the Research Papers in Economics (RePEc) database via EBSCO using the following search string:

(nonexperiment* OR non‐experiment* OR “non experiment*” OR quasi‐experiment* OR “Quasi experiment*” OR observational OR non‐random* OR nonrandom* OR “non random*” OR within‐study OR “within study” OR replicat* OR “propensity score” OR PSM or discontinuity OR RDD) AND (“experiment*” OR random*)

Snowball searches: we applied forwards citation tracking and bibliographic back‐referencing. We compiled a list of well‐known reviews of internal replication studies (Table 1). We then used three electronic tracking systems (Google Scholar, Web of Science and Scopus) to identify and screen articles that cite these reviews (forward citation tracking). We hand searched the reference lists of all primary studies in order to further identify studies that had been cited in the existing literature (bibliographic back referencing).

Institutional website repository searches: we extended our search strategy using findings from a unique project extending nearly 5 years of systematic searching, screening and indexing of impact evaluation across the field of international development. Further described by Cameron et al. (2016) and Sabet and Brown (2018), the 3ie Impact Evaluation Repository provides an index more than 4,000 impact evaluations populated through a project of systematic screening of more than 35 databases, search engines and websites. It also reports descriptive information on studies key characteristics, including study design, country of origin, sectoral focus and so forth. We use this database to identify evidence from studies in international development that are not yet recorded in the boarder internal replication literature. We screened all studies in the repository recorded as using both a randomised and nonrandomised design.

We also sought to identify literature by searching the web repository of a known producer of internal replications. Preliminary searches suggested that Mathematica Policy Research Inc. had published several internal replication studies. Therefore, we hand‐searched Mathematica's website using the search function to identify pages, documents and articles featuring the term “within‐study”.

#### 3.2.2 Data collection and analysis

We collected summary information from eligible studies on the populations, interventions, comparisons, outcomes and study designs. All studies reported treatment effects for a causal benchmark study (a sample randomly assigned in an experimental or natural experimental context), and for a nonrandomly assigned comparison replication. The replicated comparisons could be constructed using any method. We also collected outcomes data effect sizes for benchmark and nonrandomised replication study design, treatment compliance (see coding tool in A). We tabulated this information in a database format using Microsoft Excel. Finally, we used Cochrane's revised tool for RCTs ROB2.0 (Higgins, Savović, Page, & Sterne, 2016) or cluster‐RCTs (Eldridge et al., 2016) to assess biases in the benchmark RCTs.^10^

### 3.3 Search results

**Figure 1:**
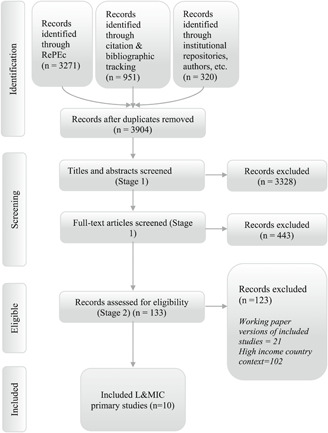
Study search flow for nonrandomised internal replication studies

Figure 1 summarises the search strategy and results for primary internal replication studies. The RePEc database search returned 3,271 records and was conducted in August 2016. Citation tracking searches, also conducted in August 2016, returned a further 951 records for screening. The search of institutional repositories, including Mathematica's (in September 2016) and 3ie's repository of impact evaluations (in July 2017), identified 307 records. Contacting authors of existing studies, and hand searches of our own personal repositories of known studies, identified 13 additional references. After removal of duplicates, a total of 3,904 records were included for screening at title and abstract.

During screening at the title and abstract 3,328 records were excluded. The remaining 576 records were screened at full text. A further 443 records were then excluded during full‐text screening, leaving 129 records which were assessed for eligibility. Of these 133 studies, 21 were removed due to being working papers of now published articles, and 102 internal replication studies were excluded due to the geographic location of the RCT not deriving from an L&MIC context. We eventually included 10 studies of social and economic programmes in L&MICs.

There were a number of excluded studies among L&MIC populations that made comparisons between randomised and nonrandomised estimates of programmes. For example, Friedman et al. (2016) were excluded because the results for the nonrandomised group were calculated using OLSs, a method which we would not expect to account for confounding satisfactorily. We similarly excluded OLS comparison group estimates from included within‐study comparisons (McKenzie et al., 2010). Other studies did not meet the required criteria to be classified as within‐study comparisons. Thus, we excluded Oosterbeek et al. (2008), Behrman et al. (2009) and Barham et al. (2014), on the basis that the populations did not overlap. Oosterbeek et al. (2008) compare the findings of a randomised experiment conducted among households with a poverty index between the 13th percentile and the 28th poverty percentile with an RDD analysis among households between the 33rd percentile and the 47th percentile. Behrman et al. (2009) provided a comparison of randomised and nonrandomised estimates using control populations with different variations in exposure to a cash transfer programme. Barham et al. (2014) compared randomised and nonrandomised estimates covering different calendar periods.

Another study by Cintina and Love (2014) also created nonrandomised treatment and control groups from an RCT by Banerjee et al. (2015), and as such, did not provide an estimate of effect of the same intervention using randomised and nonrandomised groups. Similarly, Glewwe et al. (2004) was also excluded because it formed a between‐study comparison, examining differences in effects of similar but different interventions.

### 3.4 Study descriptive information

Included studies are summarised in Table 4. Four of these studies featured in the previous review of internal replication studies in international development (Hansen et al., 2013). These are based on data from conditional cash transfer schemes, PROGRESA in Mexico (Buddelmeyer & Skoufias, 2004; Diaz & Handa, 2006) and *Red de Proteccion Social* in Nicaragua (Handa & Maluccio, 2010), and a randomised lottery balloting permanent migration visas in Tonga (McKenzie et al., 2010).^11^ One study on Mexico's cash transfer programme examines the correspondence of estimates from an RDD analysis with estimates from an RCT (Buddelmeyer & Skoufias, 2004) and we were also able to locate an additional six replications of RCTs (Barrera‐Osorio, Filmer, & McIntyre, 2014; Chaplin et al., 2017; Galiani & McEwan, 2013; Galiani, McEwan, & Quistorff, 2017; Lamadrid‐Figueroa et al., 2010; Urquieta, Angeles, Mroz, Lamadrid‐Figueroa, & Hernández, 2009).

**Table 4:** Eligible studies conducted in low‐ and middle‐income countries

Study	Intervention type	Country	Nonrandomised replication
Buddelmeyer and Skoufias (2004)	Cash Transfer	Mexico	RDD
Diaz and Handa (2006)	Cash Transfer	Mexico	Matching
Urquieta et al. (2009)	Cash Transfer	Mexico	RDD
Handa and Maluccio (2010)	Cash Transfer	Nicaragua	Matching
McKenzie et al. (2010)	Migration	Tonga	DID, Matching, IV
Lamadrid‐Figueroa et al. (2010)	Cash Transfer	Mexico	RDD
Galiani and McEwan (2013)	Cash Transfer	Honduras	RDD
Barrera‐Osorio et al (2014)	Scholarships	Cambodia	RDD
Chaplin et al. (2017)	Electrification	Tanzania	Matching
Galiani et al. (2017)	Cash Transfer	Honduras	GDD

Abbreviations: DID, difference‐in‐difference; GDD, geographical discontinuity design; RDD,regression discontinuity design.

All included studies use randomised control trials as the benchmark, with the exception of McKenzie et al. (2010) which uses a randomised natural experiment, where programme assignment was done by a public lottery by policymakers although the data itself were collected by the authors specifically to test the treatment effect. The studies test a range of nonrandomised replication methods including geographical discontinuity design (GDD), RDD, IVs, PSM and DIDs. In this section, we discuss narratively the results from the six additional internal replication studies identified in this updated search for literature. Extending the review of findings by Hansen et al. (2013), we provide a summary of the context, approach and highlights of each study.

#### 3.4.1 Lamadrid‐Figueroa et al. (2010): Impact of Oportunidades on the prevalence of contraceptive use estimated by RDD

Similar to studies described in Hansen et al. (2013), Lamadrid‐Figueroa et al. (2010) exploit the design of Mexico's randomised *Oportunidades* programme to construct “simultaneous design” internal replication study of an RDD. To provide some context, at its inception in 1997 when it was known as PROGRESA, the evaluated programme contained multiple components including conditional cash transfers, a nutrition programme and a free essential healthcare package. The distribution of the programme resources was determined by an eligibility criterion. First, a community was assessed as to whether it had sufficient healthcare facilities and schools to host the programme. Using a cluster‐randomised phase‐in design, eligible communities were then randomly assigned to begin the programme in 1997 (creating the experimental treatment group) or on a wait‐list until 2000 (forming the experimental control group). Then, at the household level, a survey of family and household characteristics determined a poverty index score for each household. Households below a set threshold were eligible to enrol on the programme, although local programme administrators did also have some discretion to influence a household's eligibility status (i.e., the household status was not strictly determined by the poverty threshold).

Lamadrid‐Figueroa et al. (2010) approximate the experimental estimates of the programme effects by differencing the randomised treatment and control community outcomes. Examining a binary outcome indicating the prevalence of contraceptive use among rural 20–24 years old in the year 2000, they report the estimated experimental ITT impacts of the programme on eligible households within participating communities.^12^ The experimental estimates are compared with nonrandomised estimates from RDD analysis examining the outcomes of observations from the eligible and noneligible households in treatment communities. Here the RDD analysis provides a localised estimate of the effects of the programme assuming the treatment assignment is “as good as random” around the eligibility threshold.

Given that a clearly defined poverty threshold determining eligibility was not available, to construct an RDD analysis Lamadrid‐Figueroa et al. (2010) first apply discriminatory analysis to ascertain a threshold poverty score that minimises the misclassification of households to the eligible group within treatment communities. They approximate 3.23% of the observations were misclassified according to this predicted cut‐off value but also use sensitivity analysis to confirm the robustness of their overall findings to alternative values. They compare observations in the treatment communities with varying windows of width around the predicted threshold values (including 50, 100 and 150 points around the cut‐off score) and the analysis reports both simple OLS estimates and 2SLS model estimates (to instrument for the possible endogeneity of the assignment of households’ eligibility status given it was not a sharp cut‐off).

The study's results show a lack of statistical correspondence between the RCT and RDD estimators. While the randomised experiment indicated that the prevalence of contraceptive use significantly increased among eligible treatment communities compared with control communities, conversely the RDD analysis estimated a large significant negative effect comparing eligible and noneligible observations. For example, the results of the randomised experiment implied the programme caused a 5% increase, on average, in contraceptive use among eligible households (*p *< .1). Meanwhile, the RDD estimates using the OLS model with the smallest window around the threshold (50 points) estimated an average decrease of approximately 22%.

The magnitude of the difference between the randomised estimate and the RDD estimate of the programme's effects decreased as the window around the threshold widened in the RDD analysis. However, the RDD estimate nevertheless remained negative, though not statistically significant, even in the specification with the largest window around the threshold (estimating a 9% decrease). The RDD 2SLS estimates provided qualitatively the same conclusions as the OLS estimates and quantitatively they were very similar in magnitude (with less than a 2% difference in estimated effects across specifications).

#### 3.4.2 Urquieta et al. (2009): Impact of Oportunidades on skilled attendance at delivery estimated by RDD

Urquieta et al. (2009) also examine the effects of *Oportunidades* on the prevalence of skilled attendants at delivery using RDD. The analysis applies the same eligibility threshold as that determined by the discriminatory analysis described in Lamadrid‐Figueroa et al. (2010). It also reports experimental ITT estimates of the effects of the programme on eligible households.^13^ However, this study limits the sample to households with at least one woman reporting a birth between 1997 and 2000 and, rather than report RDD estimates using a 2SLS specification, it controls for the potential issues of endogeneity of household eligibility status close to the threshold using a DID model on a balanced panel (i.e., among a sample of women who had births in both the baseline and the follow‐up periods). The results contrast findings from both cross‐sectional and panel data.

Using windows of 20, 30, 50, 75, 100 and 120 points around the poverty threshold, Urquieta et al. (2009) find the results of RDD analysis statistically correspond with the RCT estimates. Almost all estimates across both the RCT and RDD designs are statistically insignificant for both cross‐sectional and panel data variations of the estimates. Magnitudes of the estimates arguably also correspond between the designs. For example, coefficients of cross‐sectional randomised and RDD models imply a small effect of around 1–3% increase in skilled attendance at delivery. Meanwhile, balanced panel models for both approaches correspond to large increases in estimated effects (albeit with a high degree of imprecision), with experimental coefficients implying an 11% average increase in skilled attendance and RDD estimates ranging from 5% to 26%.

#### 3.4.3 Barrera‐Osorio et al. (2014): The effects of primary school scholarships in Cambodia on school outcomes estimated by RDD

Examining an experiment in Cambodia piloting a new government programme introducing scholarships for primary school children, Barrera‐Osorio et al. (2014) create a “simultaneous design” internal replication study. Very similar to the *Oportunidades* internal replication studies, the authors make use of a cluster‐randomised phase‐in design combined with the programme eligibility criteria to compare the estimates from a randomised experiment to those estimated by RDD.

Here schools were randomly assigned to either Phase 1 (starting in 2008/09) or Phase 2 (starting in 2009/10). The 103 schools randomly assigned to Phase 1 (the treatment group) were further randomised to either a poverty‐based scholarship system or a merit‐based scholarship system. In the poverty‐based system, the household characteristics of fourth‐grade students (third at the time of the baseline assessment) were scored by a centrally contracted firm using a poverty index to ascertain the student's poverty status. Half of the students deemed most impoverished in each school were eligible to receive the scholarship. For schools in the merit‐based system, the students with the highest baseline test scores were eligible to receive the scholarship. All scholarships equated to a value of approximately US$20 per annum and were conditional on students maintaining a certain level of attendance and grades. The remaining 104 schools randomly assigned to Phase 2 of the experiment formed the randomised control group.

Barrera‐Osorio et al. (2014) estimate the effects of the scholarships on mathematics scores and the average highest grade completed 2 years after the start of the programme. They follow the outcomes of the Grade 3 cohort assessed at baseline, reflecting that the randomised control group for this cohort (the children in Phase 2 schools) remained intact over time and did not receive the scholarships (despite their younger peers in their school becoming eligible for the scholarship in future years). In contrast to the two internal replication studies described above, they also report randomised estimates local to the threshold in addition to the randomised estimates from the broader distribution of observations. Meanwhile, the RDD analysis contrasts the results of both parametric and nonparametric econometric specifications, as well as different types of bandwidths among nonparametric estimators.

Results reported in the paper indicate that the RDD estimates are similar in magnitude to the RCT estimates but are generally less precise. For example, where the experimental results find significant positive effects on the outcome for grade completed, the RDD estimates generally do not due to the much larger standard errors. Neither the parametric or nonparametric estimators prove to consistently outperform each other. Rather, in the assessment of the poverty‐based scholarship, while nonparametric RDD estimators report higher correspondence with experimental estimates when examining math scores, the parametric estimates offer greater correspondence when examining the outcome for number of grades completed.

Comparisons of alternative types of nonparametric bandwidths find conclusions from the statistical significance of estimators are consistent with each other. However, in some instances, the reported coefficients were very different. For example, when examining grade completion outcomes in the analysis of the effects of the merit‐based scholarship, while the two nonparametric specifications using Imbens and Kalyanaraman's approach to estimating bandwidths report positive coefficients (0.25 and 0.3), the specifications using Calónico et al. (2014) approach report negative coefficients (−0.41).

#### 3.4.4 Galiani et al. (2017): Impact of conditional cash transfers on child school and labour outcomes estimated by GDD

Using a cluster‐randomised experiment in Honduras, Galiani et al. (2017) examine the effects of a conditional cash transfer programme on the probability of children enrolling in school, as well as their probabilities of working outside and inside the home. Starting in 2000, the experiment identified 70 malnourished municipalities using the 1997 census of first‐grade children's heights. Divided in quintiles, eligible households in 8 of the 14 municipalities in each quintile were then randomly assigned the cash transfers (with a proportion of municipalities also randomly receiving additional grants to schools and health centres). Households were eligible for an annual per‐child cash transfer of approximately US$50 for up to three children for children between 6 and 12 enrolled in primary school grades 1–4.

To develop another example of a “simultaneous design” internal replication study, they contrast the differences between children's outcomes from eligible households in the randomised treatment and control municipalities^14^ to estimates obtained from a geographic GDD. Here, nonrandomised comparisons are drawn comparing the outcomes of observations near to either side of a geographic boundary (which acts as a geographic discontinuity).

Given that the Honduran census does not record the precise location of dwellings, they use the latitudinal and longitudinal coordinates of *caserios* (hamlets) to determine whether a household rests within 2 km to both municipal borders and larger department borders. The latter reflects that the management and financing of public schools may vary between departments given that the centralisation of some public administrative and governance functions in Honduras. Results report robustness tests excluding households near department borders to control for this potentially confounding factor. The study also contrasts findings from the full sample of eligible households, with two limited samples, the first limited to the two “poorest” strata of households and the second limited to the third to fifth strata of households.

Overall, the results suggest that the randomised and nonrandomised GDD estimates statistically correspond, generally offering similar conclusions regarding the statistical significance of estimated effects across different samples and outcomes. More broadly, it was also concluded that the magnitude of the effects was relatively similar, although the GDD estimates were mildly smaller (downward biased). A further placebo analysis compiled a GDD analysis between the untreated nonrandomised *caserios* and randomised control *caserios* within 2 km from a municipal border. Coefficients in the placebo analysis were both very small and statistically insignificant.

#### 3.4.5 Galiani and McEwan (2013): Impact of conditional cash transfers on child school and labour outcomes estimated by RDD

Galiani et al. (2017) were based on an experiment first analysed by Galiani and McEwan (2013). Galiani and McEwan (2013) also created an RDD analysis to compare their experimental estimates. They used the nutrition‐based eligibility criteria initially imposed to select municipalities as the discontinuity in this analysis, examining the schooling outcomes of children ages 6–12 years who have not completed fourth grade residing in municipalities within (+/−) half a standard deviation of the cut‐off score imposed during municipalities selection into the RCT.

The results show that experimental results statistically correspond with the RDD analysis when examining experimental estimates for observations close to the cut‐off. Notably, RDD and RCT estimates in the vicinity of the cut‐off do not estimate significant changes in school enrolment, work outside or work inside the home. Although, despite their statistical insignificance, when examining the estimated coefficients, the correspondence between estimators is less apparent given their opposite signs.

The authors also highlight that the study offers another example where the localised treatment effect estimates taken from a nonrandomised study do not approximate ATEs estimated from a randomised experiment. Here Galiani and McEwan (2013) estimate that, on average, the cash transfers significantly increased enrolment and decrease work inside and outside the home across the treated population. However, further to internal replication studies such Lamadrid‐Figueroa et al. (2010), here the authors also show that these differences occur between the even though a degree of local correspondence exists between estimators.

#### 3.4.6 Chaplin et al. (2017): Impact of offering low‐cost electricity connection in Tanzania estimated using matching techniques

The final study we review derives a “simultaneous design” internal replication study from a cluster‐RCT introduced in 2012 in Tanzania. The experiment compares the outcomes of households from a sample of 192 communities, where 29 randomly selected communities were provided with new transmission and distribution electricity lines and the offer of low‐cost electricity connections (randomised treatment group) and the remaining 163 communities only fitted with new lines (randomised control group). Chaplin et al. (2017) form an internal replication study matching the households in randomised control communities with those from a broader group of communities not part of the programme in Tanzania.

The analysis examines 59 outcomes, covering four domains including energy use, education and child time use, business and adult time use and economic well‐being. It reports the average standardised absolute difference between the randomised and matched control groups across the 59 outcomes, as well as for each outcome domain. To form the nonrandomised comparison group, it uses two samples of matched communities from outside of the experiment. The first sample compiles a group of qualitatively matched (i.e., not statistically matched) communities with a similar proportion of households that were living in communities with new lines by the follow‐up in 2015. The second sample consists of randomly selected communities without access to electricity. This sample provides a test of the matching approach when there exists an important characteristic not accounted for in the analysis.

The analysis also contrasts the correspondence of the nonstatistical matching approach described above with statistical matching. The latter uses nearest neighbour PSM to attribute a nonrandomised comparison group to the randomised control group. It also compares the correspondence of statistical matching estimates having controlled for pretest outcomes, as well as a rich set of covariates (covering individual, household and community characteristics) and a geographically local comparison group (a community located within 40 km of the control communities). This includes comparing matching estimates having used any combination of these design elements.

The study finds that the differences between the randomised control group and matched comparison groups are, on average, statistically significant across outcomes domains. The correspondence of the matching estimator also decreases when using a low‐quality comparison sample without electricity lines (e.g., the coefficients of the average magnitude of the bias increased from 0.086 to 0.120 using the approach without statistical matching). Statistical matching generally improved the degree of correspondence between groups across outcomes and samples. In particular, statistical designs using a rich list of covariates generally increased the degree of correspondence, as did use either local geographic matching and pretest outcomes. However, matching estimators using combinations of these elements generally performed better than those using a single element and those using all three elements nearly always increased the correspondence of the matching estimator by about as much as any other combination. Statistical matching did not, however, eliminate the difference between the groups and differences were still larger when matching on communities without electricity. The latter further highlights the limitations in such statistical techniques in accounting for unobserved differences in comparison groups.

### 3.5 Risk of bias assessment in benchmark studies

Existing reviews of internal replication studies do not provide comprehensive assessments of the risk of bias to the effect estimate in the benchmark study using formal risk of bias tools. Partial exceptions are Glazerman et al. (2003), who comment on the likely validity of the benchmark RCTs (randomisation oversight, performance bias and attrition), and Chaplin et al. (2018) who code information on use of covariates to control for pre‐existing differences across groups and use of balance tests in estimation.

Our overall assessment of the risk of bias involving experiments in internal replications from L&MICs indicates that all of the experiments host low or moderate concerns. Concerns largely arise from a lack of sufficient evidence to confidently assign a “low risk” score. For instance, in some cases concerns could be alleviated with further provision of information or analysis of the underlying experiments data. For example, concerns involving the imbalances between treatment and control groups in Chaplin et al. (2017) could simply be resolved with appropriate analysis of baseline characteristics using distance metrics. Furthermore, more robustness testing and information on the sampling strategy at follow‐up involving the control group may help to alleviate some concerns with regards to missing outcomes data in McKenzie et al.'s (2010) natural experiment.

In other instances, concerns may be more difficult to address. For example, none of the studies address the issues of blinding outcome assessors and it is unknown to what extent this could influence assessments of outcomes and participant selection. Furthermore, it is challenging and rare that widely implemented social programmes such as those that feature in many of the cluster‐randomised trials assessed here can sufficiently capture data on confounding issues such as migration between clusters. This latter point may give rise to the argument that perhaps future within‐study comparisons in L&MICs would also benefit from making use of smaller and more controlled environments in order to develop internal replication studies.

Finally, a caveat of the published risk of bias tool for RCTs is that it does not provide questioning to discern the sufficiency of the application of IV estimation as a correction for noncompliance. It merely provides a decision score of “some concerns”. This means that the IV results provided by McKenzie et al. (2010) would not be able to achieve a “low risk” assessment in relation to bias arising due to deviations from intended interventions, regardless of the rigour of the analysis done.

The rest of this section states the key factors, uncertainties and decision points influencing the scores associated with each domain of bias for each experiment assessed (Table 5). It proceeds by discussing each bias domain in turn.

**Table 5:** Risk of bias assessment for benchmark experiments

	PROGRESA (Buddelmeyer and Skoufias, 2004; Diaz and Handa, 2006)	Oportunidades (Lamadrid‐Figueroa et al., 2010; Urquieta et al., 2009)	Handa and Maluccio (2010)	McKenzie et al. (2010) [IV]	Barrera‐Osorio et al. (2014)	Galiani and McEwan (2013) Galiani et al. (2017)	Chaplin et al. (2017)
Bias arising from the randomisation process	*Low risk*	*Low risk*	*Low risk*	*Low risk*	*Low risk*	*Low risk*	*Some concerns*
Bias arising from the timing of identification and recruitment of individual participants	*Low risk*	*Low risk*	*Low risk*	*NA*	*Low risk*	*Low risk*	*Some concerns*
Bias due to deviations from intended interventions	*Some concerns*	*Some concerns*	*Some concerns*	*Some concerns*	*Low risk*	*Low risk*	*Some concerns*
Bias due to missing outcome data	*Some concerns*	*Some concerns*	*Low risk*	*Some concerns*	*Low risk*	*Low risk*	*Low risk*
Bias in measurement of the outcome	*Some concerns*	*Some concerns*	*Some concerns*	*Some concerns*	*Low risk*	*Low risk*	*Some concerns*
Bias in selection of the reportedresult	*Low risk*	*Low risk*	*Low risk*	*Low risk*	*Low risk*	*Low risk*	*Low risk*
Overall bias	*Some concerns*	*Some concerns*	*Some concerns*	*Some concerns*	*Low risk*	*Low risk*	*Some concerns*

*Note*: Bias arising from timing of identification and recruitment is not assessed in individually randomised studies.

Abbreviation: NA, not applicable.

#### 3.5.1 Randomisation process and identification and recruitment of individual participants

Overall, we appraised the following studies’ randomisation processes as being of “low risk of bias” given the similarity of cluster sizes and/or balance of characteristic data (Buddelmeyer & Skoufias, 2004; Diaz & Handa, 2006; Galiani & McEwan, 2013; Handa & Maluccio, 2010; Lamadrid‐Figueroa et al., 2010; Urquieta et al., 2009).

There were some concerns in Chaplin et al. (2017) where statistical tests of the equality of means between treatment and control group baseline characteristics indicated that more frequent differences arose than would be expected by chance. Nevertheless, it is notable that even small differences may appear significant in relatively large samples (for more detailed discussion on such issues see Bruhn & McKenzie, 2009). For this reason, we consider that it would be more appropriate for the authors in these instances to analyse treatment and control group similarity using distance metrics. However, insufficient presentation of such evidence leaves us unable to conclude confidently whether the study has a low risk of bias with regards to the randomisation process (since the standard, albeit erroneous, approach is to present statistical significance testing).

With regards to assessments of the bias arising from the timing of identification and recruitment of individual participants in relation to timing of randomisation, similar issues concerning imbalance occurred when assessing Chaplin et al. (2017). Furthermore, insufficient information existed across studies relating to whether recruitment of individual participants within clusters could have been affected following cluster‐randomisation.

#### 3.5.2 Deviations from intended interventions

Deviations from the intended interventions across the cluster‐randomised studies concerned issues of implementation of the intervention in the treatment groups. For example, referring to the experiment used in Handa and Maluccio (2010), Maluccio and Flores (2004, p. 14) describe that “it was not possible to design and implement all the components according to the original timelines. In particular, the healthcare component was not initiated until June 2001… There were also delays in the payment of transfers to households due to a governmental audit that effectively froze [Red de Proteccion Social] RPS funds”. Similarly, Buddelmeyer and Skoufias (2004, p. 7) reported “in the treatment localities 27% of the total eligible population had not received any benefits by March 2000”. These findings would typically be of concern if our purpose was to generalise a statement about the effectiveness of these interventions.

However, in this instance, we are concerned with whether different methods estimate the same level of impact, regardless of whether this impact reflects a well implemented intervention or the true efficacy of the intervention or not. This argument is particularly relevant for studies such as Buddelmeyer and Skoufias (2004), Diaz and Handa (2006), Handa and Maluccio (2010), Chaplin et al. (2017) and Galiani et al. (2017) where estimates including matching the nonrandomised comparison group with the randomised control. Here we purposefully upgrade the risk of bias rating to reflect that we do not expect these issues to inflate the estimates of bias observed between experimental and nonexperimental estimates.

Nevertheless, despite having taken the purposeful decision to disregard poor implementation of the treatment itself, we still consider the cluster‐RCTs concerning cash transfers to host some concerns with regards to this bias domain. Behrman and Todd (1999 ) explain that individuals from control localities or other localities may migrate to treatment group localities in order to receive the benefits of the intervention and that the incidence of such issues should be tracked. However, in general, these studies do not indicate the extent that this issue may have occurred. Exceptions include that by Galiani et al. (2017) who highlight this as an unlikely issue in the analysed experiment. Similarly, spot checks in the experiment used for Barrera‐Osorio et al. (2014) yielded no cases of the manipulation of the scholarship selection process. According to the risk of bias tool's decision matrix, where insufficient evidence exists, the risk rating warrants a score of “some concerns”.

Finally, in the case of the natural experiment of the effects of migration on income (McKenzie et al., 2010), there is considerable noncompliance in the treatment group (i.e., a large proportion of participants randomised into the treatment group did not emigrate). Two types of experimental estimates were provided by the authors to accommodate deviations from intended interventions. These are ITT, which estimates the effect of assignment, and complier average causal effect (CACE) using IVs, measuring the effect of starting and adhering to treatment, correcting for nonrandom deviations from the intended intervention. The CACE estimate (where the randomised outcome of the random ballot is an instrument for the variable of interest—the migration decision) is the one that is incorporated in subsequent analysis and hence is presented in Table 5. An appropriate method of analysis using approaches such as IV to correct for noncompliance is scored as of “some concerns” according to the tools decision matrix.

#### 3.5.3 Missing outcome data and measurement of the outcome

With regards to bias due to missing outcome data, studies were assessed of “low risk” of bias where missing outcomes data were similar across treatment, and of “some concern” where information was not available. We score the experiment in Galiani and McEwan (2013) and Galiani et al. (2017) of “low risk” reflecting the analysis was based on census data. The study by McKenzie et al. (2010) performs purposeful sampling of the control group during the follow‐up survey because of concerns that the method of follow‐up (using a telephone directory) may lead to bias in selection into the study (for those that do not have telephones). They elect to deliberately include a sample of participants from the outer islands of Tonga in order to correct for a possible bias this may introduce. However, we remain unclear as the effect this purposeful sampling may have had on the composition of the control group and their outcome data during the follow‐up. Robustness checks and further details are not available, and therefore the study is considered to be of “some concerns”.

Across all but one experiment assessed, bias in outcomes measurement were considered to be of “some concerns”. This is largely due to the issue of lack of blinding of assessors. It is also unknown (there is insufficient evidence) to confidently state whether outcomes were likely to be influenced by knowledge of intervention received, since outcomes data were usually collected at the household level through self‐report respondent survey, rather than more rigorous methods such as formal tests.^15^ The exception is for Barrera‐Osorio, which administered mathematics and working memory tests and hence was classified as being of low risk of bias. Evidence from meta‐epidemiological studies suggests that biases in nonblinded studies are problematic when outcomes are self‐reported (Savović et al., 2012). Another exception related to the way the experiment in Galiani and McEwan (2013) and Galiani et al. (2017) was conducted. Since the data for this experiment used census data retrospectively, it is not expected that participants or assessors would associate the data collection with household treatment status. In all of these cases, we assigned “low risk of bias” in outcomes measurement.

#### 3.5.4 Selection of the reported results

Here we consider that the reporting quality generally offers low‐risk bias across the studies assessed, due to the large number of effects usually reported for different outcomes and samples. For example, all studies reported results of RCTs across multiple outcome domains, which they then used to compare with nonrandomised replications. Where particular subgroups were reported, for example, by sex in Buddelmeyer and Skoufias (2004), they were justified as common practice.

### 3.6 Quantitative estimates of bias in nonrandomised within study replications

We collected data on treatment effects for the benchmark study and each corresponding nonrandomised replication presented, from 604 specifications. These data included outcome means in treatment and control/comparison (or treatment effect estimates from an analysis), outcome variances, sample sizes, tests of statistical significance, types of variables used in adjusted analysis and available measures of treatment compliance for RCTs (see coding tool in A). We used the estimate of effect from the RCT which most closely corresponded with the population for the nonrandomised arm (e.g., the bandwidth around the treatment threshold in the case of Buddelmeyer & Skoufias, 2004). Where there was nonadherence, we used the CACE,^16^ as in the case of McKenzie (2010).

#### 3.6.1 Quantitative measures of bias

There are two main types of measures of difference between the benchmark and nonrandomised replication study arms (Steiner & Wong, 2016): distance metrics which quantify the difference between the effect size estimates between the benchmark and nonexperimental replication; and conclusion‐based measures which use information on sign, statistical significance or an effect threshold.

We calculated the standardised absolute difference between treatment effects in experimental and nonrandomised replication samples. We define *D* as the primary distance metric measuring the size of the bias between the nonexperimental and experimental results. *D*_s_ is a standardised measure of *D* which is defined in recent reviews by Wong et al. (2017) and Steiner and Wong (2016). We used the absolute difference in *D* to ensure consistency across studies reported effects; for example, Chaplin et al. (2017) only reported absolute direct standardised measures. In addition, in the subsequent results, we report averages over the large number of values of *D* collected in each study; we want a measure of the deviation of randomised and nonrandomised estimators, and not one that on average “cancels out” positive and negative deviations, hence potentially obscuring important differences. Formally *D*_s_ was computed as follows:

(1) $$\left|D_{s}\right|=\frac{\left|{\hat{\tau }}_{re}-{\hat{\tau }}_{nx}\right|}{s_{re}},\;\text{where}\;\left|{\hat{\tau }}_{re}-{\hat{\tau }}_{nx}\right|=\left|\left({\bar{Y}}_{re}^{t}-{\bar{Y}}_{re}^{c})-({\bar{Y}}_{re}^{t}-{\bar{Y}}_{nx}^{c})=({\bar{Y}}_{re}^{c}-{\bar{Y}}_{nx}^{c}\right)\right|,$$

${\bar{Y}}_{nx}^{c}\;\text{and}\;{\bar{Y}}_{re}^{c}$ are the mean outcomes of the nonexperimental comparison and experimental control groups, ${\bar{Y}}_{re}^{t}$ is the mean outcome of the experimental treatment group and *s*_re_ is the sample standard deviation of the outcome in the experimental group or the pooled standard deviation of the experimental treatment and control group.

Where an appropriate standard deviation of the outcome in the experimental group or the pooled standard deviation of the experimental treatment and control group were not reported, the standardised mean difference (*d*) for each estimator was calculated using the following formula and then subtracted from one another to compute *D*_s_, as follows:

(2) $$d_{nx}=\frac{2t}{\sqrt{n_{g}+n_{c}}},\;d_{re}=\frac{2t}{\sqrt{n_{g}+n_{c}}},\;D_{s}=|d_{re}-d_{nx}|,$$

where *n* denotes the sample size of treatment group (g) or control/comparison (*c*) and *t* is the *t*‐statistic for the difference in group means in either nonexperimental (nx) or experimental (re) samples. Where *t* was not reported, we calculated it by dividing the reported coefficient for the difference in group means by the standard error. If the authors only reported confidence intervals and no standard error we calculated the standard error from the confidence intervals (as in the case of McKenzie et al., 2010).^17^

#### 3.6.2 Comparisons of randomised and nonrandomised estimates

Reflecting issues noted by Cook et al. (2008), there are a number of strategies that within‐study comparisons have adopted in this literature to increase the similarity of randomised and nonrandomised estimators’ causal quantities. For example, with respect to RDD replication studies, Galiani and McEwan (2013), Barrera‐Osorio et al. (2014) and Galiani et al. (2017) restrict the RCT samples to create localised randomised estimates in the vicinity of the discontinuity. This approach was also previously used by Buddelmeyer and Skoufias (2004) (discussed in Hansen et al., 2013).

Alternatively, another approach used by Chaplin et al. (2017) and Galiani et al. (2017) (as well as previously by Diaz & Handa, 2006 and Handa & Maluccio, 2010) includes matching nonexperimental comparison groups with experimental control groups. Here it is assumed the ATE is theoretically zero given that the control group is not exposed to the treatment. Any differences that then arise between the two matched groups is attributed to an inconsistency between estimators.

We extracted data relating to estimates using such strategies to minimise the differences in causal quantities between experimental and quasi‐experimental strategies. However, such estimates are not available in studies by Urquieta et al. (2009) and Lamadrid‐Figueroa et al. (2010). We report the available estimates of bias reported in these studies for completeness.

#### 3.6.3 Quantitative estimates of bias

We calculated bias estimates for all included studies and report the mean standardised bias in Table 6. These bias estimates are the simple averages from 604 individual standardised absolute mean differences of bias and their standard errors. The results show the bias estimates are usually small, meaning frequently <0.1 standard deviations in the outcome and often not significantly different from zero. Given that the benchmark experiments were assessed as being of low or moderate risk of bias, this suggests that the methods used to design and implement nonrandomised internal replications in L&MICs may yield treatment effect estimates that are close to the true effect for the particular sample.

**Table 6:** Standardised bias estimates in internal replication studies in L&MICs

Study	NRS type	Mean bias	95% confidence interval		No. bias estimates
Barrera‐Osorio et al. (2014)	RDD	0.061	−1.687	1.809	20
Buddelmeyer and Skoufias (2004)	RDD	0.026	−0.044	0.097	324
Chaplin et al. (2017)	Matching	0.058	**0.037**	**0.079**	16
Diaz and Handa (2006)	Matching	0.036	**0.006**	**0.066**	24
Galiani and McEwan (2013)	RDD	0.011	−0.598	0.619	9
Galiani et al. (2017)	GDD	0.009	−0.290	0.308	27
Handa and Maluccio (2010)	Matching	0.433	**0.377**	**0.489**	132
Lamadrid‐Figueroa et al. (2010)^a^	RDD	0.130	−0.100	0.360	6
McKenzie et al. (2010)	IV, DID, Matching	0.127	−0.268	0.522	22
	*IV*	*0.110*	−*1.61*	*1.83*	*3*
	*DID*	*0.206*	−*0.10*	*0.51*	*2*
	*Matching*	*0.120*	−*0.04*	*0.28*	*18*
Urquieta et al. (2009)^a^	RDD	0.058	−0.168	0.284	24

*Note*: Confidence interval in bold indicates *p* < 0.05; reported bias estimates are taken using experimental estimates reporting similar causal values to nonexperimental estimates (if available).

Abbreviations: DID, difference‐in‐difference; GDD, geographical discontinuity design; RDD, regression discontinuity design.

^a^ Denotes study using bias estimates not using similar causal values.

Three of the four studies using nonrandomised matching estimators report statistically significant average differences (Chaplin et al., 2017; Diaz & Handa, 2006; Handa & Maluccio, 2010). One set of estimates from Handa and Maluccio (2010) are relatively large on average (0.43 standard deviations), but within this study the bias coefficients greater than one standard deviation reflect some of the estimates contained in that study with weaker matching strategies (those not involving a combination of geographical and household level variables). In the same study, preferred matching strategies—where matching selected geographically proximate and similar households—yielded an average bias estimate across expenditure and health outcomes of 0.03 (95% confidence interval [−0.02, 0.08]).

Other than Handa and Maluccio (2010), two studies report average estimates >0.1 standard deviations. While neither are statistically significant, Lamadrid‐Figueroa et al. (2010) and McKenzie et al. (2010), respectively, report average estimates of 0.130 and 0.127. Here we note that Lamadrid‐Figueroa et al. (2010) does not provide experimental and nonexperimental causal values that are similar. The relatively large estimates and standard errors from McKenzie et al. (2010) may also reflect issues relating to the benchmark experiment. Both our critical appraisal and discussions in previous reviews by Cook et al. (2008) and Hansen et al. (2013) highlight some concerns about that study's potential risk of bias.

A final point of note is warranted regarding the discontinuity designs. All of the studies examining discontinuity designs that use equivalent causal estimands (i.e., with the exception of Lamadrid‐Figueroa et al., 2010), produce distance metrics that are small on average (<0.06 standard deviations). None are significantly different from the benchmark estimate. However, because of the local population around the cut‐off over which discontinuity designs are estimated, they are also of low power which would account for the statistically insignificant findings; for example, Goldberger (1972) originally estimated sampling variances for an early conception of RDD as being 2.75 times larger than an RCT of equivalent sample size. Presumably, this is also the case of estimates from other designs that produce global (rather than local) causal estimands which would explain why some of the findings from matching estimators are of similar small magnitude as the RDD estimates, but significantly different from zero at standard significance levels.

## 4 RISK OF BIAS IN RDD

High quality systematic reviews set explicit study design inclusion criteria, and then transparently appraise included studies based on the quality in which they are designed and implemented (internal validity) and the relevance of the evidence for decision making (external validity; Higgins & Green, 2011; The Steering Group of the Campbell Collaboration, 2014; Waddington et al., 2012). In systematic reviews examining questions about the effects of programmes on outcomes, assessment of internal validity is done in “risk of bias” assessment. Risk of bias tools provide the criteria to enable reviewers to evaluate transparently the likelihood of bias, for particular bias domains (e.g. confounding, selection bias, deviations from intended interventions, bias in outcomes data collection and reporting). A recent review paper (Waddington et al., 2017) argues that the existing risk of bias tools are, to differing degrees, insufficiently conceptualised to assess bias for nonrandomised studies of interventions commonly used by social scientists, including RDD. That paper, along with Bärnighausen, Oldenburg, et al. (2017), discusses the assumptions underlying different nonrandomised quasi‐experimental methods, on which risk of bias tools may usefully draw. The main complications of non‐randomised studies, including a priori credible designs with selection on unobservables like RDD, are the greater assumptions and need for diagnostic tests making them “more susceptible to influence from researcher expectations and hypotheses that can bias study results towards what is expected or desired rather than what is true” (Chaplin et al., 2018, p.7).

In the following section, we present results of a review of approaches used in risk of bias assessment in Campbell reviews, including RDDs. Subsequently, we present an approach to conducting risk of bias assessment in RDDs.

### 4.1 Risk of bias in Campbell systematic reviews

We downloaded records from the Campbell Library and collected the following data:

- Study information: Lead coordinating group and study identifiers (lead author and year).
- Study inclusion criterion: Whether the review eligibility criteria included nonrandomised studies of effects.
- Incorporation of RDD: Whether the criteria for the review included RDDs, whether any were found and included, and whether any were excluded.
- Risk of bias approach: The tools used to evaluate risk of bias and the bias domains reported in the results.

We reviewed all 99 Campbell systematic review reports published between January 2012 and December 2018, of which 80 (81%) included nonrandomised studies.^18^ All reviews published by Education Coordinating Group (ECG) and International Development Coordinating Group (IDCG; including Nutrition Group) incorporated nonrandomised studies. Furthermore, 83% of the Crime and Justice Coordinating Group (CJG) and 55% of Social Welfare Group (SWG) reviews incorporated nonrandomised studies. In one Knowledge Translation and Implementation Group review, nonrandomised studies were included as eligible but none were found (Petkovic et al., 2018). Authors used different tools to assess risk of bias for included nonrandomised studies (Table 7), roughly corresponding to the group coordinating the registration process. SWG authors mainly used either an early version of Cochrane's risk of bias tool for nonrandomised studies of interventions (Reeves, Deeks, Higgins, & Wells, 2011), or Cochrane's risk of bias tool for RCTs (Higgins et al., 2011), as did half of ECG reviews. IDCG authors largely used the tool developed by 3ie (sometimes attributed to IDCG, other times as Hombrados & Waddington, 2012), although two used the tool developed by Cochrane Effective Practice and Organisation of Care (EPOC, [Link]), and one used Cochrane's Risk of Bias in Nonrandomised Studies of Interventions (ROBINS‐I; Sterne et al., 2016; known as ACROBAT at the time the reviews were undertaken). CJG authors mainly used tools they developed for the specific purposes of the review in question.

**Table 7:** Main risk of bias tools used to assess nonrandomised studies by lead group

	CJG	ECG	IDCG	SWG	Total
3ie (Hombrados & Waddington, 2012)	–	–	9	1	10
Cochrane EPOC (n.d.)	–	–	3	–	2
Higgins et al. (2011)	1	8	2	7	13
ROBINS‐I (Sterne et al., 2016)	–	1	1	–	1
EPHPP (n.d.)	–	–	1	–	1
Reeves et al. (2011)	–	2	–	10	8
Other	11	7	4	–	15
None	2	1	1	–	2

*Note*: ‐ indicates no reviews used this tool. Some reviews use multiple tools.

Abbreviations: CJG, Crime and Justice Coordinating Group; ECG, Education Coordinating Group; IDCG, International Development Coordinating Group; SWG, Social Welfare Group.

We also collected data on the domains of bias contained in the tools used to assess studies incorporated in Campbell reviews. We focused on bias domains considered particularly relevant for RDDs: confounding and selection of the reported result (as defined in Sterne et al., 2014). 'Confounding bias' occurs when factors which predict the outcome also determine receipt of intervention. This includes selection to intervention by participants (e.g. on the basis of need) or practitioners (e.g. on the basis of eligibility), or programme placement bias by planners (e.g. on the basis of geographical unit). Confounding can be observed or unobserved (unmeasured or unmeasurable), time‐invariant (fixed over the course of the study at baseline) or time‐varying. Some types of confounding bias can be controlled in analysis, for example observables can be controlled in adjusted analysis, and time‐invariant confounding (including unobservables) can be controlled through statistical modelling (e.g. difference‐in‐differences analysis of pre‐test and post‐test outcomes between intervention groups in order to account for), and most effectively in study designs which in theory can control for unobservable and observable confounders (e.g. RCTs and RDDs). We found that 94 percent of Campbell reviews incorporating non‐randomised studies assessed risk of bias due to confounding. 'Bias in selection of the reported result' may refer to outcomes or analysis, and may be addressed through reporting of pre‐analysis plans, transparent reporting of any analyses that were determined post hoc, and reporting on all outcomes and participant sub‐groups measured irrespective of findings. Over 80 percent (83%) of Campbell reviews incorporating non‐randomised studies specifically assessed bias in selection of the reported result. Other sources of bias defined by Sterne et al. (2014) which are relevant for RDDs are not covered here because the signalling required to answer them are unlikely to be different for RDDs as for other designs (e.g. 'performance bias', is when participants receive a different intervention to the one intended, and 'bias in outcomes data collection' due to measurement error in outcomes reported by participants or measured by outcomes assessors). One source of bias, 'bias in classification of interventions', was deemed irrelevant for RDDs, because assignment to intervention is based on a score measured at pre‐test. RDDs are sometimes eligible for, and included in, Campbell systematic reviews. In 2012–2018, 35 reviews explicitly allowed for the inclusion of RDD (51% of reviews that incorporate nonrandomised studies, or 35% of all reviews)—that is they mention “regression discontinuity” explicitly in the study inclusion criteria, or define inclusion that would be consistent with RDD (e.g., allocation by “time differences, location differences, decision‐makers, or policy rules”; Filges, Jonassen, & Jørgensen, 2018, p. 19). Twelve reviews were able to include at least one RDD in analysis (18% of reviews incorporating nonrandomised studies; Table 8). These include eight reviews in international development, which draws on a cross‐disciplinary literature including econometrics where the method has become popular, plus three reviews in social welfare and one in education. In a few instances, authors differentiate types of discontinuity design such as Lawry et al. (2014) and Samii et al. (2014) who each include one GDD, and Piza et al. (2016) and Filges et al. (2018) who include “fuzzy” RDDs. In other cases, however, RDDs were excluded from analysis. Petrosino et al. (2012) differentiated experimental and observational designs, including two prospective RDDs and excluding two others that are natural experiments (defined by the authors as “investigator initiated not based on actual program rules”, p. 100). Turner et al. (2018, p. 16) note that “[a]lthough Regression Discontinuity Designs (RDDs)… generate data that can be used to make causal inferences, they were excluded from this review because statistical methods for incorporating RDD… data into meta‐analyses are, to the best of our knowledge, not well‐established”.

**Table 8:** Inclusion of RDD in Campbell reviews

	RDD eligible	RDD included	RDD excluded
CJG	3	0	0
ECG	9	1	1
IDCG	20	8	2
SWG	3	3	0
Total	35	12	3

Abbreviations: CJG, Crime and Justice Coordinating Group; ECG, Education Coordinating Group; IDCG, International Development Coordinating Group; RDD, regression discontinuity design; SWG, Social Welfare Group.

Previous research has found that existing risk of bias tools are not operationalised to assess nonrandomised studies with selection on unobservables (Waddington et al., 2017). This is partly because most tools were not developed to address particular designs like RDDs. For example, we are aware of four tools that present signalling questions for RDDs (Chief Evaluation Office, undated; Hombrados & Waddington, 2012; Schochet et al., 2010; Valentine & Cooper, 2008). The tools on which RDDs have been critically appraised in Campbell reviews are Hombrados and Waddington (2012), Reeves et al. (2011) and Higgins et al. (2011).

### 4.2 Definitions of RDDs

Given the increased interest in RDD as a method of programme evaluation, the incorporation of these studies in systematic reviews and concerns about methods for their synthesis, we elected to focus our work here on RDD. Using a mixture of literature review (of existing risk of bias tools, textbooks and recent journal articles) and expert consultation, we developed signalling questions under seven bias domains.

**Figure 2:**
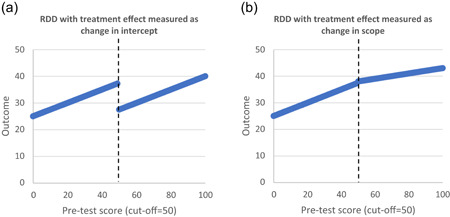
Examples of RDD

RDD, also called RD and “cut‐off based design”, was conceived of as in educational psychology by Thistlethwaite and Campbell (1960) (cited in Shadish et al., 2002) and in economics by Goldberger (1972). In RDD, assignment to treatment is based on a specific score on an ordinal or continuous measure, such as a diagnostic test, that is given prior to treatment. For example, an educational authority might choose to administer a math test to all 10 year‐old students, and provide extra support for students scoring below some threshold of proficiency. Students just on either side of the cut‐off threshold should be highly similar to one another, and therefore if there is a treatment effect this should be noted in a discontinuity (or break) in outcomes between the treated and untreated groups at the point of intervention, which may be a change in intercept or slope, or a combination. Figure 2 presents two simple examples of the relationships between the assignment variable (pretest score with cut‐off set at 50) and outcomes; there are many more possibilities allowing for nonlinear relationships between assignment variable and outcome (see, e.g., Shadish et al., 2002). RDD has become a popular approach to treatment effect estimation,^19^ particularly in economics. Key review articles and practice guidelines are available in econometrics (Imbens & Lemieux, 2007; Lee & Lemieux, 2010), educational psychology (see e.g., Cook, 2008) and, latterly, epidemiology (Moscoe, Bor, & Bärnighausen, 2015).

We give several definitions of the RDD approach by leading authors in Table 9. The definition by Shadish et al. (2002) implies that it is the experimenter who designs the study prospectively. However, discontinuity assignment may also arise from natural processes of policy and practice—for example, age requirements for pensions and biometric tests used in medicine. Where outcomes data are available, or can be collected, this opens up the approach to retrospective analysis provided data are also available on the assignment variable used to allocate units prehoc; in other words, RDD as a natural experiment (Dunning, 2012).

**Table 9:** Definitions of regression discontinuity by key authors

Shadish et al. (2002): “The experimenter assigns units to conditions on the basis of a cutoff score on an assignment variable… The assignment variable can be any measure taken before treatment, in which the units scoring on one side of the cutoff are assigned to one condition and those on the other side to another” (p. 208)
Schochet et al. (2010): “A study qualifies as an RD study if… treatment assignments are based on a forcing variable [which] must be ordinal with a sufficient number of unique values” (pp. 2–3)
Dunning (2012): “Individuals or other units are sometimes assigned to the treatment or control groups according to whether they are above or below a given threshold on some covariate or pretest. For units very near the threshold, the process that determines treatment assignment may be as good as random, ensuring that these units will be similar with respect to potential confounders” (pp. 63–64)
Cook (2014): “The regression discontinuity design (RDD) occurs when assignment to treatment depends deterministically on a quantified score on some continuous assignment variable. The score is then used as a covariate in a regression of outcome. When RDD is perfectly implemented, the selection process is fully observed and so can be modelled to produce an unbiased causal inference” (p. 1)

Cook's (2014) definition as presented here suggests a deterministic relationship between the assignment variable and treatment status, but Cook also refers to “fuzzy” RDD which allows for a probabilistic relationship between assignment and treatment (in other words, there are additional factors, which may or may not be measured, affecting assignment status). The quote from Dunning (2012) also indicates that, at least in social science research and econometrics, the causal relationship is usually estimated close to the cut‐off threshold. However, the global relationship may also be estimable under stronger assumptions (Angrist & Rokkanen, 2015; Bor, Moscoe, & Bärnighausen, 2015; Bor, Moscoe, Mutevedzi, Newell, & Bärnighausen, 2014; Rubin, 1977).^20^ Finally, Schochet et al. (2010) give guidance on the nature of the assignment variable, suggesting scope for ordinal scales to be included with sufficient units either side of the threshold.

In theory, RDD generates an unbiased treatment effect. This is because the assignment rule is known, hence the relationship between treatment and outcomes can be modelled with trivially small confounding, at least close to the assignment threshold (Cook, 2008). Its closest design relative is the RCT.^21^ In economics, political science and many health applications (see Dunning, 2012; Moscoe et al., 2015), RDD has frequently been used retrospectively to evaluate existing threshold rules. Because RDD needs large samples, it is well suited to retrospective evaluation using existing data sources (e.g., administrative data).

Lee and Lemieux (2010) identified 67 RDD studies in economics between 1983 and 2009. Dunning (2012) reviews 22 examples of RDD natural experiments in social science research between 1999 and 2009. According to the literature review by Moscoe et al. (2015), there were—at time of writing—32 examples of RDD to assess the impact of interventions on health outcomes in PubMed. Most of the RDDs found evaluated social and economic interventions. Only two evaluated the effects of clinical interventions: Almond et al. (2010) evaluating health care programmes assigned by birth weight, and Bor et al. (2015) evaluating antiretroviral therapy assigned by CD4 count. A forthcoming systematic review covering health, social science and grey literature by Hilton‐Boon et al. (2016) identified 177 RDDs published before March 2015 evaluating the effect of an intervention or exposure on a physical or mental health outcome. This indicates the great scope and practice of RDDs being undertaken in health and economics alone.

### 4.3 Methods of assignment and analysis in RDD

In RDD, treatment is assigned ex‐ante according to a known rule—specifically, a threshold on a scale variable measured among participating units at pretest. Units scoring on one side of the threshold subsequently receive treatment, while those on the other do not. The treatment effect is estimated by comparing observations from different units observed contemporaneously, immediately on either side of the threshold. Different types of assignment variables have been used in RDD analyses (Dunning, 2012; Hahn, Todd, & van der Klaauw, 2001; Moscoe et al., 2015) including but not limited to:

- Test scores: for example, continuous biomarkers in medicine (e.g., cholesterol, blood glucose, birth weight, CD4 count); entrance exams in education.
- Programme eligibility criteria: for example, poverty index; criminality index.
- Age‐based thresholds: for example, voting age; pension age; birth date/quarter.
- Size‐based thresholds: for example, hospital size; school size.
- Geographical threshold (GDD): for example, administrative boundary.
- Time (RD in time [RDiT]): for example, date of a policy or practice change.

In GDD, exposure to the treatment depends on the position of observations with respect to an administrative or territorial boundary (e.g., Galiani et al., 2017). In the particular case where the assignment variable is time, RDiT is similar to controlled ITS (Hausman & Rapson, 2018; Shadish et al., 2002). Perhaps one difference between ITS and RDiT is that treated and untreated samples are different, which makes a difference to the length of follow‐up period over which treatment effects can be credibly estimated. In ITS, the same participating units are followed‐up over time, and the treatment effect is identified through variation in exposure to treatment over time, sometimes with respect to an untreated comparison (Somers, Zhu, Jacob, & Bloom, 2013). It is most credible in estimating treatment effects for observations immediately after the time of intervention in comparison with their values immediately before (i.e. the short term effect). In contrast, in RDD, the treatment effect is estimated by comparing observations from different units measured at the same time (or follow‐up period); the comparison is made up of units who were eligible immediately before or after a threshold date on which a policy or practice change occurs. However, the outcome for those units could be assessed many years later.

**Figure 3:**
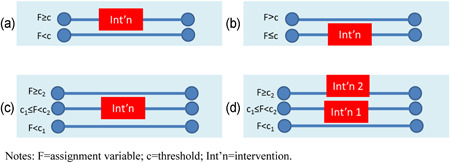
Examples of assignment rules used in RDD. RDD, regression discontinuity design

In the basic design, assignment to treatment and comparison is based on the observational unit's pretest score on the continuum, relative to the assignment threshold (Figure 3)—whether above the threshold (panel a) or below it (panel b). Variants on the design might include multiple thresholds on the assignment continuum within which treatment is assigned (Table 10, panel c), or multiple thresholds for different levels of treatment (panel d; Shadish et al., 2002). While the forcing variable is necessarily measured at pretest, outcomes may only be available at posttest.

**Table 10:** Proposed signalling questions for risk of bias in regression discontinuity design: Confounding and selection of the reported result (modifications highlighted in bold font)

Definition of study designs/analysis types to be addressed				
**RDD**		**A study that estimates an intervention effect by allocating participants to treatment and comparison groups on the basis of a threshold on a scale variable measured at pretest, and comparing outcomes among units either side of the threshold at post‐test. Examples of threshold variables include: Test scores such as continuous biomarkers (e.g., cholesterol, blood glucose, birth weight, CD4 count) or entrance exams in education; age‐based thresholds (e.g., pension age); size‐based thresholds (e.g., class size); or time (e.g., date of birth, date of policy or practice change)**		
		**RDD may be designed prospectively where outcomes data are collected by experimenters at baseline and endline specifically for the purposes of evaluating the intervention. However, it is common for RDD studies to be conceived and analysed retrospectively, for example, by using routine data collection. In both instances, participants are allocated to intervention at pretest**		
**Notes on RDD approaches**				
**RDD shares many similarities with RCTs. While assignment to intervention is not random (an observation's location on a scale variable either side of a threshold is used to determine assignment), estimation methods are used to identify ‘as‐if random’ allocation at the threshold** **Similar to RCTs, the main threat to internal validity for RDD is manipulation of the value of the threshold variable by participants and practitioners. That is, nonrule‐based discretionary assignment by practitioner, rather than rule‐based assignment determined centrally, or if there is public knowledge of the assignment mechanism and participants are able to manipulate assignment variable score to determine access. However, in the case of RDD, causal identification can also rest on random error in measurement of the assignment variable at the threshold** **The main difference between RDD and RCT is in the treatment assignment mechanism, which then implies different analytic techniques to estimate the causal effect.** Shadish et al. (2012) **list the following main threats to estimation of the causal effect in an RDD: Nonlinear relationship between assignment variable and outcome (requiring use of correct polynomial functions of assignment variables), interactions with a third variable causing the relationship between assignment and outcome to be probabilistic rather than deterministic (so‐called ‘fuzzy RDD’), or where variables are non‐normally distributed** **A threat to internal validity arises if the same threshold value is used for assignment to interventions, other than the intervention of interest, which may affect the outcomes of interest (‘treatment confounding’). In such a case, the threshold variable cannot be used to estimate the causal effect of the intervention of interest, but it can be used to estimate the effect of the combined interventions** **RDDs can estimate the LATE directly around the threshold and, under much stronger assumptions, the (global) ATE that would be estimated in an RCT for the sample population** **In RDD, the relationship between assignment variable and treatment status may be deterministic (sometimes known as ‘sharp RDD’) or probabilistic (sometimes known as ‘fuzzy RDD’), in which case factors other than the threshold rule influence treatment assignment. Under ‘fuzzy RDD’ IV estimation should be used to estimate the treatment effect (known as the complier average causal effect, CACE)**				
	***The effect of interest***			
	**The treatment effect may be estimated as a change in intercept and/or slope, and is estimated as either:**			
	**The difference in outcomes (intent‐to‐treat effect) at the threshold, also called local average treatment effect (LATE or, alternatively, average treatment effect at the cut‐off)** **The difference in outcomes (intent‐to‐treat effect) across the entire distribution, also called (global) average treatment effect (ATE), which can be obtained via extrapolation in RDD under additional assumptions** **The difference in outcomes among “compliers”, that is, units induced to take up the treatment because of the threshold rule (complier average causal effect, CACE at the threshold), estimated by instrumental variables regression**			
**1. Bias due to confounding**				
Signalling questions			Description	Response options
**1.1 Is the relationship between the assignment variable (a continuous variable or a discrete scaled variable with sufficient points either side of the cut‐off) and outcome demonstrated?**			**Demonstration should be done using a graph of the assignment‐outcome relationship. Appropriate functional form may include local linear regression at assignment threshold, or ordered polynomial. The treatment effect may be measured as a change in intercept and/or change in slope (regression‐kink design)**	Y/PY/PN/N
**1.2 Was treatment assigned in an unbiased way?**			**Unbiased assignment means that classification of intervention status is not affected by systematic manipulation of the assignment variable by participants or decision makers:**	Y/PY/PN/N
			a) **The assignment decision rule should be adequately concealed from participants, or** b) **assignment variable nonmanipulable by participants, practitioners or other decision makers, or** c) **the assignment variable should be measured with random error**	
			**The study should describe methods of allocation concealment, or argue convincingly for randomness in measurement error. Where the assignment variable is continuous, the study should report a histogram of the assignment variable to demonstrate that bunching does not occur around the threshold. Where the assignment variable is discrete, McCrary or Frandsen tests are more conclusive**	
**1.3 Is the relationship between assignment variable and outcomes unconfounded at the threshold?**			**Support for this can be obtained by assessing:**	Y/PY/PN/N
			**The distributions of baseline characteristics above and below the cut‐off** **Addition of a phase in which intervention is not present, for example, by estimating the preintervention relationship between assignment variable and outcomes at baseline, or is removed (and possibly further phases where it is added again). Responsiveness of the outcome variable to these changes in intervention can help verify the functional form and to adjust for nonlinearities in the relationship** **Addition of a nonequivalent outcome, or ‘placebo outcome’. That is, assessing the effect on a second outcome variable that the intervention should not influence, as a falsification exercise** **‘Placebo discontinuity’ tests showing no other discontinuities in the assignment variable within the bandwidth of interest, as a falsification exercise**	
			**If the assignment rule is also used to allocate another intervention (“treatment confounding”), which may affect the outcomes of interest, then the method can only be used to estimate the effect of the combined interventions, and cannot be used to isolate the effect of the intervention of interest**	
**2. Bias in selection of the reported result**				
2.1. Is the reported effect estimate likely to be selected, on the basis of the results, from multiple outcome *measurements* within the outcome domain?			For a specified outcome domain, it is possible to generate multiple effect estimates for different measurements. If multiple measurements were made, but only one or a subset is reported, there is a risk of selective reporting on the basis of results	Y/PY/PN/N/NI
2.2 Is the reported effect estimate likely to be selected, on the basis of the results, multiple analyses of the intervention‐outcome relationship?			**Researchers should analyse the change in slope and/or level using different band‐widths around the threshold or functional form. The following should be prespecified as far as possible, and reported in sensitivity analysis:**	Y/PY/PN/N/NI
			**Selection of optimal bandwidth using existing data‐driven routines;** **Selection of appropriate functional form for the relationship between assignment and outcome variables; and** **Robustness checks to other bandwidths and functional form specifications**	
2.3 Is the reported effect estimate likely to be selected, on the basis of the results, from different *subgroups*?			Particularly with large cohorts often available from routine data sources, it is possible to generate multiple effect estimates for different subgroups or simply to omit varying proportions of the original cohort. If multiple estimates are generated but only one or a subset is reported, there is a risk of selective reporting on the basis of results	Y/PY/PN/N

*Note*: Suggested changes for RDD indicated in bold font. All other text taken from Sterne et al. (2014). Only bias domains 1 (confounding) and 7 (bias in selection of the reported result) are shown here. Other bias domains are relevant for RDD ‐ but are not shown here as changes to existing ROBINS‐I signalling questions are not being suggested at this point.

Abbreviations: ATE, average treatment effect; LATE, local average treatment effect; RCT, randomised assignment design; RDD, regression discontinuity design.

In the simplest formulation of the RDD, the treatment effect is estimated as the change in the intercept at the threshold in a linear regression of outcome measured at posttest on the assignment variable (Figure 2). The treatment effect is estimated as the vertical difference between the regression line for the observed outcome for treated units, and the extrapolated counterfactual outcome from the regression line for controls.

Estimating the correct functional form of the relationships between assignment variable and outcome is the main estimation concern. Researchers must correctly establish the functional form to avoid a potentially confounded relationship—for example, where a linear regression line is fitted to a nonlinear relationship (Angrist & Pischke, 2009, p. 254; Shadish et al., 2002). Variants on the estimation approach include allowing the treatment effect to manifest as a change in slope as well as, or instead of, a change in intercept (by incorporating interaction terms in regression estimation). For example, in the “regression‐kink design” the treatment effect is estimated as the change in slope of the outcome variable at the threshold (Figure 2).

The counterfactual approach, and the modelling of functional form, can be strengthened by data. Availability of pretest outcomes data can strengthen the approach. The clear advantage of prospectively designed RDDs—over and above the advantage they have over retrospective studies in controlling for selection bias into the study—is that outcomes can be measured before assignment across participants, allowing estimation of the relationship between assignment variable and outcomes among different units at pretest. This can verify the counterfactual relationship at pretest for observations before they are assigned, in order to confirm the functional form. Where treatment occurs among units in time‐dependent batches, there may be scope to estimate the counterfactual functional form relationship at the same time among different groups (Cook, 2008).^22^ Recruitment into the study of a “pure control” group that is subjected to the same informed consent and data collection also has the advantage of enabling measurement of motivation biases in prospective studies (see below). The addition of a nonequivalent dependent variable function (“placebo outcome”) can also be added to provide a “no‐cause counterfactual” (Trochim, 1984; cited in Cook, 2008). Tests for “placebo discontinuity” at different thresholds of the assignment variable can help rule out the existence of a chance relationship with the outcome of interest (Moscoe et al., 2015).

Most RDDs are analysed retrospectively using routine data collection. This has implications for the analysis that can be done to verify underlying assumptions of the approach (e.g., limits to testing the preintervention relationship between assignment variable and where outcomes baseline data are not available). But there may also be advantages of retrospectively conducted RDDs over prospective designs. One clear advantage of retrospective studies in general is that participants are not aware they are part of a research study at the time of intervention. Hence, threats to validity arising in prospective studies due to behavioural responses to being observed in treatment groups (Hawthorne effects) or control groups (compensatory rivalry [John Henry] or resentful demoralisation; Bärnighausen, Tugwell, et al., 2017b), or social desirability bias from outcomes data that are self‐reported rather than directly observed (Schmidt, 2014), may be less relevant. A disadvantage of retrospective RDD is that one really needs to know a lot about the situation in which RDD is implemented to judge its validity—for example, whether placement scores could be manipulated (by selectively allowing some people to retake the placement test).

### 4.4 Internal validity in RDD

The main threats to internal validity in RDD concern inappropriate characterisation of the assignment variable or the relationship between assignment variable and outcome, and manipulation by participants or implementers of the assignment variable.

The assignment variable should be a scale variable with sufficient unique values. Usually, this would be a continuous variable, although the What Works Clearinghouse Standards for RDD states the minimum requirement is for an ordinal scale with four units either side of the treatment threshold (Schochet et al., 2010).^23^ The assignment variable does not need to be truly continuous, provided it is a scaled variable with sufficient possible values either side of the threshold in order for a credible linear line between the assignment variable and the outcome to be estimated.

In order for RDD to produce internally valid estimates, the minimum criterion is exchangeability at the threshold—that is, the potential outcomes would be same on average if treated units had been untreated and untreated individuals had been treated, as would be the case in a well‐conducted RCT. One common way that this is violated is if the assignment variable itself is precisely manipulabled by participants or implementers, at least over the subsample of observations around the cut‐off threshold. Threats to validity may arise where there is public knowledge among programme participants of a manipulable assignment variable, or where practitioners are able to assign to treatment on a discretionary basis. This is equivalent to assessing subversion of randomisation when random allocation is not concealed until after recruitment in RCTs. Hence, participants should either be blinded to the value of their assignment variable or unable to manipulate it (and practitioners should not be involved in assignment, or will not be able to manipulate it). Assignment variables which participants have manipulated include reported income, which may be incorrectly reported or manipulated to gain eligibility to programmes (Buddelmeyer & Skoufias, 2004).

In addition, the relationship between the assignment variable and outcome must be unconfounded over the sample in which the treatment effect is estimated. The most important cause of confounding is manipulation by participants or planners. This assumption is easiest to establish for the sample of observations closest to the threshold where there is random error in measurement of the assignment variable (Goldberger, 1972). Where an assignment rule determines eligibility for more than one intervention affecting the outcome of interest (treatment confounding), it will not be suitable for RDD analysis of the effects of the intervention of interest (Schochet et al., 2010). An example is evaluations of school lunch programmes in which family income is used for programme eligibility at the same cut‐off as is used for other state welfare benefits. This is the same as a violation of the exclusion restriction in an IVs set up, or the selection‐by‐history effect that could also occur in an RCT. However, it still offers a valid opportunity to assess the overall ITT effect of assignment to the full package of interventions.

Hence three causal estimands can be distinguished, relating to assumptions about homogeneity of treatment effect across the full sample distribution:

1. Where treatment effects are variable across the distribution, RDD is able to estimate the LATE (also called the ATE at the cut‐off; Hahn et al., 2001). In the simplest case, where there is random noise in the measurement of a (nonmanipulable) assignment variable, units immediately around the threshold can be considered as randomly allocated to treatment and control (Goldberger, 1972). Where the assignment variable is measured without noise, a stronger assumption is needed, that is, that the relationship between the assignment variable and outcome is unconfounded in the region of the threshold. Another way of saying this is that the potential outcomes (expected outcomes conditional on the assignment variable) are assumed to be continuous at the threshold (Bor et al., 2015; Hahn et al., 2001).
2. Where treatment effects are constant across the assignment variable distribution (i.e., slope of assignment–outcome relationship independent of assignment variable), RDD can estimate the ATE. In this case, the functional form of the relationship between assignment variable and outcome must be modelled appropriately over the full distribution of the assignment variable. Strong assumptions are needed for identification, such as the relationship between assignment and outcome being unconfounded over the entire distribution of the assignment variable (potential outcomes are continuous across the full distribution; Bor et al., 2015).
3. Where the relationship between assignment variable and outcome is probabilistic rather than deterministic (“fuzzy RD”), IVs is used to estimate the CACE.

### 4.5 Statistical conclusion validity in RDD

The main factors affecting the validity sources of a statistical test of the treatment effect in RDD arise from: Choice of bandwidth around threshold, estimation of the functional form and adherence to assignment rule.

Statistical modelling to determine the sample over which to estimate the treatment effect is crucial in RDD. Under the weaker validity assumption for LATE, this means determining the appropriate bandwidth around the threshold for local linear regression, which is usually done using covariate balance tests to examine the assumption that groups on either side of the threshold are roughly equivalent in their baseline characteristics (Schochet et al., 2010).

Estimating the correct functional form of the forcing assignment variable is the main estimation concern. As noted above, the relationship between outcome and assignment variable may be nonlinear and confound the treatment effect estimate. There are two ways to estimate the relationship:

1. Parametric approaches: The use of parametric approaches does not necessarily imply that the relation is linear. Ideally one should try and report different polynomial orders for the assignment variable, and then (using Akaike Information Criteria and visual inspection) identify the preferred specification under different bandwidths of the assignment variable.
2. Nonparametric approaches: These do not make any assumptions about functional form. For this reason, Imbens and Kalyanaraman (2012) argue that this technique is preferable. The key issue when using this approach is in defining the optimal bandwidth. The state of the art option used by econometricians is the optimal bandwidth procedure defined by Calónico et al. (2014).

We can distinguish two forms of nonadherence in RDD analysis, which affect the estimation methods used: Nonadherence to the assignment rule; and fuzziness (cf. misclassification) in the relationship between assignment variable and assignment status. We note here that these are conceptually different from manipulation of the forcing variable, which is a major threat to validity in RDD, as noted above.

Imbens and Lemieux (2007) and Cook (2008), citing Trochim (1984) distinguish “sharp” and “fuzzy” RDD. In the case of a “sharp” RDD, the relationship between assignment variables and treatment status is known and deterministic. In analysis, this means there will be no value of the assignment variable at which different units may be both treated and untreated. There may still be overrides to the assignment due to nonadherence, which can be addressed in ITT analysis.

This is a qualitatively different case from fuzzy RDD, where the assignment scale only determines the probability of treatment status, for example because additional factors are taken into account to determine assignment. The distinction is between fuzziness in the assignment threshold value, as in the case of overrides to the assignment rule in sharp RDD, versus allowing for other factors to determine treatment uptake, with the assignment cut‐off fixed, in fuzzy RDD. In fuzzy RDD there are elements of the rule determining assignment which may be “unknown” and in analysis, there may be values of assignment in which different units may be both treated and untreated, hence the need for two‐stage least squares estimation, where RDD assignment is used as an IV.

Other implementation problems, such as nonrandom attrition and other sources of incomplete data, and biases in outcome measurement, can be assessed using standard approaches.

### 4.6 Reporting standards and a preliminary risk of bias tool

There is potential for bias if the assumptions for RDD are not met, or not demonstrated and reported to be met. For example, adequate reporting of the assignment mechanism is vital in RDD. Lee and Lemieux (2010) and Moscoe et al. (2015) discuss reporting criteria for RDD. We list the following as being important in justifying the approach:

1. Clear presentation of the assignment–outcome relationship in a graph showing the discontinuity. Appropriate functional form may include local linear regression at assignment threshold, or ordered polynomial. The treatment effect may be measured as a change in intercept and/or change in slope (regression‐kink design).
2. Discussion of the validity conditions in the context of the study, particularly around manipulation of assignment variable score, demonstrating that: the assignment decision rule was adequately concealed from participants; the assignment variable was nonmanipulable by participants, practitioners or other decision makers; or the assignment variable was measured with random error.
3. Confirmation tests:

- Reporting the distributions of baseline characteristics above and below the cut‐off.
- Histogram of assignment variable demonstrating no data bunching around threshold, hence no manipulation of treatment status.
- Addition of a phase in which intervention is not present (e.g., by estimating the preintervention relationship between assignment variable and outcomes at baseline), or among a “pure control” group that is not offered treatment assignment, to verify functional form and to adjust for nonlinearities in the relationship.

4. Falsification tests:

- Analysis of “placebo discontinuities” at different thresholds of the forcing variable showing no other discontinuities in the assignment variable within the window of interest.
- Addition of a nonequivalent outcome, or “placebo outcome”. That is, assessing the effect on a second outcome variable that the intervention should not influence, as a falsification exercise.

5. Reporting multiple specifications to check robustness. This includes testing the robustness of the results to the use of: nonparametric methods using different bandwidths; and parametric methods using different windows for the assignment variables and different polynomial orders when modelling the relation between assignment and outcome variables.
6. Demonstration that there is no “treatment confounding”: that is, the allocation rule is only used to assign interventions of interest, and not additional interventions which may affect the outcomes of interest in the study.
7. In the case of prospectively designed RDD (QEs), bias in selection of participants into the study can be addressed by controlling for baseline imbalances, similar to RCTs. Bias in outcomes measurement may be minimised by use of methods to account for biases, such as blinding of intervention or measurement of outcomes using direct observation rather than participant self‐report.
8. In the case of retrospectively designed RDD drawing on observational datasets (natural experiments), bias in selection of participants into the study may be problematic, but bias in outcomes measurement (e.g. due to self‐reported outcomes) less so.

On the basis of this review, we propose signalling questions within relevant bias domains of ROBINS‐I. ROBINS‐I is grouped around seven bias domains as explained further in Sterne et al. (2016). Here we propose modifications (highlighted in bold in Table 10) for two domains: bias due to confounding, and bias in selection of the reported result.

Risk of bias due to confounding includes questions about the definition of the assignment scale (continuous or discrete), the specification of the relationship between assignment and outcome, treatment confounding and the assessment of balance. Thus we might expect credible RDDs to: use a continuous variable for assignment; use an appropriate method to examine the relationship with outcomes (e.g., nonparametric kernel regressions) as well as report sensitivity analysis; report a graph of the discontinuity to show no other discontinuities in the assignment variable within the window of interest; report a histogram (kernel density plot) of the assignment variable to spot bunching around the threshold which might be indicative of manipulation; and report baseline data to assess the preintervention relationship. Some of these reporting requirements, such as graphing the discontinuity, have only become common in RDD reporting latterly. For reporting bias, we may expect authors to present multiple findings for all outcomes prespecified and including multiple bandwidths.

## 5 DISCUSSION AND OPPORTUNITIES FOR FURTHER RESEARCH

The main results of this paper have shown that a wide range of risk of bias tools and internal study replications exist and may be drawn on in designing future internal study replications and risk of bias tools. In particular, drawing on a specialised and more in‐depth systematic search of impact evaluations from international development, it highlights that a much larger body of evidence exists in this field than was previously known. Here the review identifies that at least 10 within study comparisons from international development exist, more than doubling the number previously reviewed. We consider that these studies can be used in further developing the accuracy of bias assessments and provide the grounds on which they can be evidenced.

A synthesis of findings from primary studies and existing reviews of internal study replications from international development has highlighted that nonrandomised estimators can provide estimates very similar to randomised estimates, but that they do not always eliminate bias. In particular, evidence suggests that reductions in bias may occur under conditions where the outcome or selection process can be simply or credibly modelled and when estimators use a richer degree of contextual and unit information. However, studies such as Lamadrid‐Figueroa et al. (2010) and Galiani and McEwan (2013) also empirically demonstrate that randomised and nonrandomised estimators can provide very different results given the sensitivity of to the distributional effects of programmes. This point has numerous important implications. First, this highlights that during a synthesis of a literature, differences in findings between randomised and nonrandomised designs evaluating similar programmes should not necessarily be attributed to study bias. Second, it highlights the importance of experimental evidence looking beyond ATEs (examining the heterogenous effects of programmes). Third, it also empirically supports previous discussion by Cook et al. (2008) noting the need for internal replications to improve the similarity of the causal quantities reported by different estimators.

In this review, we have observed a number of strategies applied in internal replications featured in the international development literature to increase the similarity of randomised and nonrandomised estimators’ causal quantities. For example, studies used subsamples of randomised treatment groups or matching nonexperimental comparison groups with experimental control groups. We note that another innovative example featuring in the literature outside of international development includes applying transformations to estimators to increase their comparability. Gill et al. (2016) develop a method for converting nonexperimental estimators into an estimate that can be compared with experimental ITT estimates. This point highlights an interesting opportunity for future research in internal replication studies to further engage: comparing the findings of different techniques for adjusting estimators.

Despite the growth of the internal replications in international development, the total number of studies remains relatively small. This provides an impetus to continue to grow this existing body of evidence with new applications and studies. However, as highlighted in arguments by Smith and Todd (2005), future research would benefit from exploring the implications of context, as well as different statistical applications. Further research understanding whether aggregate conditions and target populations effect estimators bias seems intuitively of high importance to international development, which often works in very diverse contexts with specific and marginalised populations. Other examples of factors could relate to understanding the implications of different methods of survey administration for bias and the management of missing data. This reflects the context of limited resourcing driving demand for cost‐effective methods of data collection and common issues of low levels of recorded data in many L&MICs.

Further research would also benefit from exploring whether specification tests can be used to “rule out” biased estimates. For instance, assessments by Heckman and Hotz (1989) of the predictive accuracy of diagnostics tests of pre‐programme alignment of participant's characteristics first showed promise in helping to improve our ability to detect a biased estimator. Findings from Glazerman et al. (2003) further indicated that specification tests can help to eliminate the poorest performing non‐randomised estimates. To date, the internal replication literature from L&MICs provides relatively little analysis to contribute to the understanding of the efficacy of diagnostic tests. In some cases, the L&MIC studies do use diagnostic tests—for example, Chaplin et al. (2017) assess the balance of variables of in their final propensity score models. However, these studies do not evidence whether such diagnostics can be used to predict consistently when biased estimators will occur.

Examples of more contemporary diagnostic tests that could also be considered include whether researchers can accurately detect sensitivity of nonrandomised estimators to unobserved biases (e.g., see Arceneaux, Gerber, & Green, 2010).

Finally, this review has further highlighted the need for risk of bias approaches to be further developed for other credible nonrandomised methods, due to the increasing use of these approaches including QEs and natural experiments in programme evaluation, as well as their incorporation in systematic reviews. Here we have suggested development of an approach for RD. Further work should take place to pilot this approach and, importantly, develop algorithms for reaching transparent “risk of bias assessments” for particular domains. Further work is also needed on other approaches, for example, statistical matching approaches and DIDs, popular methods of programme evaluation for which careful risk of bias assessment is not commonly undertaken in systematic reviews. This work can also draw on existing empirical studies of robustness (e.g., Dong & Lipsey, 2014; Rosenbaum, 2010).

Correspondingly, future research related to reviews of internal replication studies could consider an updated method‐orientated review of nonrandomised matching studies in this literature. Chaplin et al. (2018) serve as an example of such a study on RDD. Other methods that may warrant reviews include panel regressions (such as DID), selection models, nonrandomised IVs and ITS analysis. We also note that the results of this study highlight that broader efforts to identify all existing internal replication studies should consider more specialised systematic search strategies within particular literatures (so to overcome the issue of a lack of systematic indexing of this evidence). With respect to international development, future updates of this review would be particularly warranted as systematic searches on databases such as the 3ie impact evaluation repository continue to be updated and expanded.

## CONTRIBUTIONS OF AUTHORS

H. W. developed the proposal and coordinated the work. H. W. and P. F. V. developed the study protocol. P. F. V. and H. W. conducted the searches and P. F. V., Chris Coffey and H. W. collected data from included studies. P. F. V. did the critical appraisals and reviewed the within study comparison literature, with inputs from H. W. H. W. did the literature review of regression discontinuity designs and developed the risk of bias tool. Julian Higgins, Jeff Valentine and Sebastian Vollmer provided technical oversight for the project as a whole. H. W. and P. F. V. wrote the final report.

## DECLARATIONS OF INTEREST

The authors are not aware of any financial or other interests which might have influenced the work undertaken.

## Footnotes

1 Some authors (Dunning, 2012) differentiate natural experiments from QEs. In these conceptwualisations, natural experiments are defined as those applying statistical techniques to observational data sets using knowledge about natural processes of programme assignment (e.g., policy, geography) to generate “as good as randomised” assignment. These are therefore retrospective observational designs with selection on unobservables. Quasi‐experiments comprise the remaining nonrandomised studies that are prospective in design (i.e., the data are collected by researchers for the explicit purpose of evaluating the intervention; Shadish et al., 2002).

2 Long‐term effects may be difficult to measure in field experiments due to contamination of controls, especially in phased‐in designs, and difficulties in locating participants in long‐term follow‐ups.

3 Although not commonly argued in Campbell reviews, nonrandomised evidence may also be incorporated in systematic reviews to document unintended or adverse effects since, the argument goes, such studies do not require strong counterfactual designs (for an example from medicine, see Golder, Loke, & Bland, 2011).

4 Where the nonrandomised study uses a credible design or method to control for confounding, thus yielding an unbiased estimate for the underlying sample, any difference in effect size with the randomised study will be due to sampling error, which would tend to cancel out on average in expectation in meta‐analysis.

5 The programmes included Mexico's PROGRESA and Nicaragua's *Red de Proteccion Social*.

6 The visas enabled Tongans permanent residency in New Zealand under the Pacific Access Category (PAC; New Zealand's immigration policy which allows an annual quota of Tongans to migrate).

7 Four studies addressed the same intervention—the US National Supported Work Demonstration.

8 In some instances, approximations calculated differences equivalent to approximately 10% of annual earnings for a typical population of disadvantaged United States workers.

9 The rationale for including quasi‐experimental approaches with selection on observables, but excluding regression with adjustment for observable confounding, is that the former methods at least balance covariates and therefore account for biases arising from comparing observationally dissimilar groups (Heckman, Ichimura, & Todd, 1997).

10 We considered possibilities of blinding coders by removing the numeric results and the descriptions of results (including relevant text from abstract and conclusion), as well as any identifying items such as author's names, study titles, year of study and details of publication. We were not able to undertake this blinding under the project due to the team's existing knowledge about the included studies.

11 The visas enabled Tongans permanent residency in New Zealand under the PAC (New Zealand's immigration policy which allows an annual quota of Tongans to migrate).

12 Lamadrid‐Figueroa et al. (2010) also provide randomised estimates of Oportunidades effects in a broader community sample (including noneligible households). This estimate was found to be positive but statistically insignificant. We focus on comparing the experimental effects estimated for eligible households.

13 Again, RCT results are reported for communities (including noneligible households). Here we focus on randomised results of the effects of the programme on eligible households.

14 Randomised experimental estimates are also provided for broader samples that do not share a municipal border with untreated nonexperimental *caserios* and that are located >2 km from a municipal border. Here we simply focus our attention on the experimental estimates using the sample most comparable to that used in the GDD analysis (i.e., those that do share a boarder and are <2 km away).

15 In some instances, outcomes were collected at community level, for example, Chaplin et al. (2017) household electricity grid connections, but these were not used in the within‐study comparison.

16 CACE may be estimated using IVs (Angrist, Imbens, & Rubin, 1996) or calculated by deflating ITT using available information on adherence (Bloom, 2006). For example, if nonadherence *C* in the treatment group is observed at a rate of 10%, for a homogenous effect (*E*) of an intervention that equates to a particular increase (say 5%) in a given outcome of interest, we would expect the ITT estimator to report a smaller average effect. This would be calculated as follows: *E*_ITT_ = *E*_TOT_(1 −* C*) = 5*(0.9) = 4.5%.

17 A second type of distance metric is a simpler measure of the percent difference (Steiner & Wong, 2016; as also reported in McKenzie et al., 2010), but is not synthesised across studies in this report. This expresses the difference between experimental and nonexperimental effect estimates as a percent of the absolute value of the experimental estimate (Steiner & Wong, 2016). However, as highlighted by Steiner and Wong (2016) the distances can be extremely large if the experimental estimate is close to zero. Formally, the measure can be computed as follows: $D_{P}=\frac{{\hat{\tau }}_{nx}-{\hat{\tau }}_{re}}{\left|{\hat{\tau }}_{re}\right|}\times 100$.

18 Nonrandomised studies are defined here as approaches in which assignment to intervention is based on some method other than randomised or quasi‐randomised (e.g., alternation) assignment.

19 Hits on “regression discontinuity” in Google Scholar (ran December 21, 2017): up to 1999, 632; 2000–2009, 3,920; 2010 to present, over 17,000.

20 Angrist and Rokkanen (2015) focus on reweighting internally valid local effects to other values of the assignment variable based on the distribution of other pretreatment characteristics.

21 The combined RDD and RCT design assigns units to intervention randomly, when the units fall within a range of scores on an eligibility variable (Shadish et al., 2002).

22 These methods may also be available in retrospectively designed RDDs, where baseline and/or control group data are available.

23 Peter Schochet in personal communication stated: “the issue about the scoring variable having ‘at least four unique values below the cutoff and four unique values above the cutoff’ was determined by an expert panel as a reasonable approximation for defining a ‘continuous’ scoring variable… What is ‘continuous’ will clearly depend on the context”. Cochrane's Effective Practice and Organisation of Care (EPOC) group also used a minimum approach to define ITS studies as having at least three observations before and after intervention, on the intuition that a minimum of three observations are required to identify a trend.

## APPENDIX A SYSTEMATIC REVIEW CODING TOOL

### Section 1 (S1): Study, Intervention And Estimate

1. Record ID: Study Unique ID number [Open Answer]
2. Record subcode: To be used for studies with more than one intervention, unit = alphabet, for example, a, b, c [Open Answer]
3. Estimate ID: To be used when extracting more than one set of comparison estimates from a study [Open Answer]

### Section 2 (S2) Study Details

1. Title: [Open Answer]
2. Authors: State author surnames, for example, Diamond and Sekhon or Bratberg, Grasdal, and Risa [Open Answer]
3. Year of publication/release: [Open Answer]
4. Publisher: for example, Journal of Development Effectiveness [Open Answer]
5. Publication Type: [Choice: peer‐reviewed article, book or chapter; contractor report to government or foundation; working paper; Conference proceedings]

### Section 3 (S3): Intervention Details

1. Intervention name: [If applicable, if not just describe the type of intervention in a few words] [Open Answer]
2. Description of intervention: Brief description of intervention as given by study [Open Answer]
3. Intervention category: [edit according to intervention focus]
4. Programme Mandatory: If S3.Q3 = Employment, Jobs and welfare, state whether the programme was mandatory [Binary: 1 = Yes, No = 0]
5. Education Level: If S3.Q3 = Education state what level of education the intervention focuses on [Choice: Pre‐school; Elementary/Middle School; Post‐Secondary]
6. Country: Country intervention originates from [Open Answer]
7. L&MIC: Is the country stated in S2.Q6 a low or middle income country (World Bank classifications) [Binary: 1 = Yes, 0 = No]
8. Was the intervention implemented nationally [Binary: 1 = Yes, 0 = No]
9. Non‐national Interventions: If S2.Q8 = 0, provide details of geographic focus of intervention—for example which states implemented in. [Open Answer]
10. Intervention Location: [Choice: Single site; Multisite, Single state; Multistate]

### Section 4 (S4): Study Characteristics

1. Outcome definition: Description of outcome from study [Open Answer]
2. Outcome Category:
3. Outcome Type: [Choice: Binary; Continuous; Nominal; Ordinal etc.]
4. Outcome Standardised: Has the outcome variable been standardised [Binary: Yes = 1, No = 0]
5. Dataset: Does the dataset derive from an existing within‐study comparison (is the data being re‐used to formulate a new analysis) (note: this does not include study's where only one element such as the RCT has been previously published) [Binary: Yes = 1, No = 0]
6. Data Origin: State the authors, year of release and title of the article that the data derives from [Open Answer]
7. Sample: If study uses multiple samples (or subsamples) provide brief description of sample for corresponding the estimates, for example, girls between 12 and 16 years of age [Open Answer]
8. Years of data collection: State years of data collection for relevant estimate, including preintervention and baseline if applicable. Indicate baseline year. E.g where there are three preintervention years (1997‐2000) and 2001 indicates the first follow up year: 1998; 1999; 2000 [Baseline]; 2001; 2002; 2003 [Open Answer]
9. Follow‐up N: Calculate number of years between baseline (the intervention start date) and the final year of data collection [Open Answer]
10. Follow‐up points: How many data points exist between baseline and endline (include endline), for example, using the example from S4.Q7, answer = 3 [Open Answer]
11. Preintervention points: How many data points exist between the first preintervention data point and baseline (include first point and baseline), for example, using the example from S4.Q7, answer = 3 [Open Answer]
12. Unit: Describe the unit of analysis, for example, individual, household, school, village [Open Answer]

### Section 5 (S5): Within‐study comparison method

1. WSC Description: Description of how comparison groups created from study [Open Answer]
2. WSC Method: State the type of within‐study comparison being used (see Table 8 for definitions). [Choice: Independent WSC; Synthetic Design; Simultaneous Design; Multisite Simultaneous Design]
3. Comparison Origin: Did the comparison group (nonrandomised control group) derive from a national dataset/survey [Binary: 1 = Yes, 0 = No]
4. Geographic Match: Was the comparison group drawn from the same labour market, school district, or relevant geographic area [Choice: same specific area (e.g., same labour market or school); same general area (e.g., same state or school district); not matched geographically

### Section 6 (S6): Benchmark (experimental) analytical approach

1. EX Method Type: Describe the method used to compute the experimental benchmark estimate [Choice: Use Cross‐sectional regression; DID; Fixed Effects; IV; Other; Combination of methods]
2. EX Method Type Combination: If S6.Q1 = Combination of methods, describe combination using choice options from S6.Q1 as closely as possible. [Open Answer]
3. EX Method Type Other: If S6.Q1 = Other, define/describe model type [Open Answer]
4. EX Model Type: Define the model used to compute the benchmark experimental estimate [Choice: OLS; Probit; Logit; Other]
5. EX Model Type Other: If S6.Q4 = Other, define/describe model type [Open Answer]
6. EX IV: If S6.Q1 = IV, define/describe instrument used [Open Answer]
7. EX Method Notes: Further notes [Open Answer]
8. EX covariate adjustment used: Were covariates used in estimating programme impacts (i.e., were they regression adjusted?) [Binary: 1 = Yes, 0 = No]
9. EX covariate adjustment number: How many were used [Open Answer]
10. EX covariate adjustment description: List the covariates used [Open Answer]
11. EX Estimator: What treatment effect is estimated [Open Answer]

### Section 7 (S7): Nonexperimental analytical approach

1. NX Method Type: Define the method used to compute the NX estimate [Choice: Statistical Matching; DID; RD; Fixed Effects; Selection Model; ITS; IV; Weighting Methods; Cross‐sectional Regression; Other; Combination of methods]
2. NX Method Type Combination: If S7.Q1 = Combination of methods, describe combination using choice options from S7.Q1 as closely as possible. [Open Answer]
3. NX Method Type Other: If S7.Q1 = Other, define/describe model type [Open Answer]
4. NX Model Type: Define the model used to compute the NX estimate [Choice: OLS; Probit; Logit; Other]
5. NX Model Type Other: If S7.Q4 = Other, define/describe model type [Open Answer]
6. Matching Method: If S7.Q1 = Statistical Matching, define type of matching [Choice: Nearest Neighbour Matching; Kernel Matching; Mahalanobis Matching; Abadie and Imbens Metric Matching; Euclidean Metric Matching; Coarsened Exact Matching; Radius Matching; Strata/Interval Matching; Covariate Matching; Support Vector Machine Matching; Genetic Matching; Random Recursive Partitioning; Optimal Matching; Other]
7. Matching Method Other: If S7.Q6 = Other, define/describe matching method [Open Answer]
8. Matching Method One‐to: If S7.Q1 = Statistical Matching, if applicable – is matching method using one to one or one to many matching [Choice: one to one; one to many; not applicable]
9. Matching Method Trimming: If S7.Q1 = Statistical Matching, is sample trimming being performed [Binary: 1 = Yes, 0 = No]
10. Matching Method Trimming Interval: If S7.Q9 = Yes, what is the trimming interval if available [Open Answer]
11. Matching Method Calipers: If S7.Q1 = Statistical Matching, is the analysis using calipers [Binary: 1 = Yes, 0 = No]
12. Matching Method Caliper Width: If S7.Q11 = Yes, state the caliper width if available [Open Answer]
13. RDD Parametric: If S7.Q1 = RD, is the model parametric [Binary: 1 = Yes, 0 = No]
14. RDD Function: If S7.Q13 = No, state the function being applied in the nonparametric model, for example, Uniform; Quartic; Guassian and so forth. [Open Answer]
15. RDD Bandwidth: If S7.Q1 = RD, state size of bandwidth [Open Answer]
16. RDD Caliper: If S7.Q1 = RD, describe if uses Caliper [Open Answer]
17. NX IV: If S7.Q1 = IV, define/describe instrument used [Open Answer]
18. ITS Pre Points: If S7.Q1 = ITS, state how many preintervention data points have been used [Open Answer]
19. ITS Post Points: If S7.Q1 = ITS, state how many post‐intervention data points have been used [Open Answer]
20. ITS Group: If S7.Q1 = ITS, is the analysis a single group or multi‐group ITS [Open Answer]
21. ITS Autocorrelation: If S7.Q1 = ITS Describe whether/how the analysis corrects for autocorrelation
22. Weighting Method: If S7.Q1 = Weighting Method, state the weight method used [Choice; Inverse Probability Weight; Propensity Score Weighting; Entropy Balancing; Other]
23. Weighting Method Other: If S7.Q21 = Other, define/describe the method used [Open Answer]
24. Selection: ADD QUESTIONS ABOUT SELECTION MODELS! [TBC]
25. NX Method Notes: Further notes [Open Answer]
26. NX Estimator: What treatment effect is estimated [Choice: ITT; ATE; LATE]

### Section 8 (A): Benchmark effect size data extraction

1. EX Pre Mean T: What is the pretreatment mean for the outcome variable in the Treatment group [Open Answer]
2. EX Pre Mean C: What is the pretreatment mean for the outcome variable in the Control group [Open Answer]
3. EX Pre Mean Pooled: What is the pooled pretreatment mean for the outcome variable
4. EX Post Mean T: What is the post‐treatment mean for the outcome variable in the Treatment group [Open Answer]
5. EX Post Mean C: What is the post‐treatment mean for the outcome variable in the Control group [Open Answer]
6. EX Pre Std T: What is the pretreatment standard deviation for the outcome variable in the Treatment group [Open Answer]
7. EX Pre Std C: What is the pretreatment standard deviation for the outcome variable in the Control group [Open Answer]
8. EX Pre Std Pooled: What is the pooled pretreatment standard deviation for the outcome variable [Open Answer]
9. EX Post Std T: What is the post‐treatment standard deviation for the outcome variable in the Treatment group [Open Answer]
10. EX Post Std C: What is the post‐treatment standard deviation for the outcome variable in the Control group [Open Answer]
11. EX Pre N T: What is the pretreatment sample size for the Treatment group [Open Answer]
12. EX Pre N C: What is the pretreatment sample size for the Control group [Open Answer]
13. EX Pre Total N: What is the total sample size pretreatment
14. EX Post N T: What is the post‐treatment sample size for the Treatment group [Open Answer]
15. EX Post N C: What is the post‐treatment sample size for the Control group [Open Answer]
16. EX Post Total N: What is the total sample size post‐treatment (Treatment group and Control group combined) [Open Answer]
17. EX Cluster N T: How many clusters are there in the Treatment group (if applicable) [Open Answer]
18. EX Cluster N C: How many clusters are there in the Control group (if applicable) [Open Answer]
19. EX Cluster Total N: How many clusters are there in total (Treatment group and Control group combined)
20. EX Cluster Unit: Define the unit of clusters. For example, locality [Open Answer]
21. EX Coefficient: What is the experimental coefficient estimate [Open Answer]
22. EX T‐stat: What is the corresponding T‐statistic for the coefficient reported in S8.Q21 [Open Answer]
23. EX P‐value: What is the corresponding P‐value for the coefficient reported in S8.Q21 [Open Answer]
24. EX Std Error: What is the corresponding standard error for the coefficient reported in S8.Q21 [Open Answer]
25. EX Std Error Info: Describe any extra information regarding standard errors in S8.Q24. For example, Robust standard errors, clustered errors, bootstrapped errors and so forth. [Open Answer]
26. EX Data References: Describe which pages, tables, rows and columns the data in Section 8 has been extracted from. [Open Answer]

### Section 8 (B): NX effect size data extraction

1. NX Pre Mean T: What is the pretreatment mean for the outcome variable in the Treatment group [Open Answer]
2. NX Pre Mean C: What is the pretreatment mean for the outcome variable in the Control group [Open Answer]
3. NX Pre Mean Pooled: What is the pooled pretreatment mean for the outcome variable
4. NX Post Mean T: What is the post‐treatment mean for the outcome variable in the Treatment group [Open Answer]
5. NX Post Mean C: What is the post‐treatment mean for the outcome variable in the Control group [Open Answer]
6. NX Pre Std T: What is the pretreatment standard deviation for the outcome variable in the Treatment group [Open Answer]
7. NX Pre Std C: What is the pretreatment standard deviation for the outcome variable in the Control group [Open Answer]
8. NX Pre Std Pooled: What is the pooled pretreatment standard deviation for the outcome variable [Open Answer]
9. NX Post Mean Std T: What is the post‐treatment standard deviation for the outcome variable in the Treatment group [Open Answer]
10. NX Post Mean Std C: What is the post‐treatment standard deviation for the outcome variable in the Control group [Open Answer]
11. NX Pre N T: What is the pretreatment sample size for the Treatment group [Open Answer]
12. NX Pre N C: What is the pretreatment sample size for the Control group [Open Answer]
13. NX Pre Total N: What is the total sample size pretreatment
14. NX Post N T: What is the post‐treatment sample size for the Treatment group [Open Answer]
15. NX Post N C: What is the post‐treatment sample size for the Control group [Open Answer]
16. NX Post Total N: What is the total sample size post‐treatment (Treatment group and Control group combined) [Open Answer]
17. NX Cluster N T: How many clusters are there in the Treatment group (if applicable) [Open Answer]
18. NX Cluster N C: How many clusters are there in the Control group (if applicable) [Open Answer]
19. NX Cluster Total N: How many clusters are there in total (Treatment group and Control group combined)
20. NX Cluster Unit: Define the unit of clusters. For example, locality [Open Answer]
21. NX Coefficient: What is the nonexperimental coefficient estimate [Open Answer]
22. NX T‐stat: What is the corresponding T‐statistic for the coefficient reported in S9.Q21 [Open Answer]
23. NX P‐value: What is the corresponding P‐value for the coefficient reported in S9.Q21 [Open Answer]
24. NX Std Error: What is the corresponding standard error for the coefficient reported in S9.Q21 [Open Answer]
25. NX Std Error Info: Describe any extra information regarding standard errors in S9.Q24. For example, Robust standard errors, clustered errors, bootstrapped errors and so forth. [Open Answer]
26. NX Data References: Describe which pages, tables, rows and columns the data in Section 9 has been extracted from. [Open Answer]
